# Calibration and Inter-Unit Consistency Assessment of an Electrochemical Sensor System Using Machine Learning

**DOI:** 10.3390/s24134110

**Published:** 2024-06-25

**Authors:** Ioannis D. Apostolopoulos, Silas Androulakis, Panayiotis Kalkavouras, George Fouskas, Spyros N. Pandis

**Affiliations:** 1 Institute of Chemical Engineering Sciences (ICE-HT), Foundation for Research and Technology Hellas (FORTH), 26504 Patras, Greece; japostol@iceht.forth.gr (I.D.A.); silas.androul@gmail.com (S.A.);; 2 Department of Chemical Engineering, University of Patras, 26504 Patras, Greece; 3 Institute for Environmental Research & Sustainable Development, National Observatory of Athens, 11810 Athens, Greece; 4 Department of Environment, University of the Aegean, 81400 Mytilene, Greece

**Keywords:** machine learning, electrochemical sensors, air quality

## Abstract

This paper addresses the challenges of calibrating low-cost electrochemical sensor systems for air quality monitoring. The proliferation of pollutants in the atmosphere necessitates efficient monitoring systems, and low-cost sensors offer a promising solution. However, issues such as drift, cross-sensitivity, and inter-unit consistency have raised concerns about their accuracy and reliability. The study explores the following three calibration methods for converting sensor signals to concentration measurements: utilizing manufacturer-provided equations, incorporating machine learning (ML) algorithms, and directly applying ML to voltage signals. Experiments were performed in three urban sites in Greece. High-end instrumentation provided the reference concentrations for training and evaluation of the model. The results reveal that utilizing voltage signals instead of the manufacturer’s calibration equations diminishes variability among identical sensors. Moreover, the latter approach enhances calibration efficiency for CO, NO, NO_2_, and O_3_ sensors while incorporating voltage signals from all sensors in the ML algorithm, taking advantage of cross-sensitivity to improve calibration performance. The Random Forest ML algorithm is a promising solution for calibrating similar devices for use in urban areas.

## 1. Introduction

Industrialization, urbanization, and vehicular emissions have led to the proliferation of pollutants in the atmosphere, necessitating the development of efficient monitoring systems to assess air quality levels [1,2,3]. Traditional air quality monitoring networks rely on expensive, accurate instruments, limiting their deployment and scalability, particularly in resource-constrained regions. The emergence of low-cost air quality sensor systems presents a promising solution to this challenge [4]. These sensors offer a cost-effective alternative to conventional instruments, enabling widespread deployment [5,6,7,8,9,10,11]. Despite their affordability and portability, the accuracy and reliability of these sensors have often been questioned, primarily due to issues such as variability across sensor units, drift [5,6,8,12,13,14], cross-sensitivity [4,5,6,7,15,16,17,18,19,20,21,22], dependence on environmental conditions [6,14,21,22,23,24,25], and ageing [7,26].

Various techniques, including machine learning (ML) algorithms and other methods, have been used to calibrate low-cost air quality sensor systems. By continuously monitoring sensor outputs and correlating them with reference data from high-precision instruments, ML models can effectively learn and adjust for drift patterns, cross-sensitivities, and temperature sensitivity, thereby maintaining the accuracy of sensor readings over extended periods [5,6,27].

Despite their potential benefits, all calibration approaches also face certain challenges. One notable limitation is the issue of transferability, wherein calibration models developed for specific sensor configurations or environmental conditions may not apply to different settings or sensor types [6,13,22,28]. This lack of generalizability necessitates careful validation and adaptation of such models to ensure their effectiveness across diverse applications and operating conditions. Another limitation, frequently underestimated by practitioners, is that of inter-unit consistency. The latter issue is usually encountered in electrochemical sensor systems, wherein the output voltages vary among sensors. Calibration methods often fail to account for this variability, introducing uncertainty in the estimated concentrations. 

This paper focuses on the calibration and inter-unit consistency assessment of an electrochemical sensor system entitled ENSENSIA (Environment Sensing Appliance) [27,29]. In its present configuration, ENSENSIA measures carbon dioxide, nitric oxide, nitrogen dioxide, ozone, fine particle matter, and total organic volatile compounds. It also provides environmental conditions (temperature and relative humidity sensor). 

The paper explores three potential calibration methods for converting the electronic signal of four air quality sensors (CO, NO, NO_2_, and O_3_) to concentrations. The first method relies on the voltage-to-concentration conversion provided by the sensors’ manufacturer. The second approach applies an additional computational step involving ML, which uses the collective sensors’ concentrations (as computed by the first approach) to improve the precision. The third approach involves ML applied directly to the sensors’ voltage signals and using the individual sensors’ electronic coefficients.

Unlike previous efforts that predominantly focused on calibration against high-cost reference equipment without addressing inter-unit variability, this work suggests the utilization of ML to minimize the variability between identical sensors. Specifically, we implement and compare calibration techniques that not only convert voltage signals to pollutant concentrations but also enhance the inter-sensor consistency that is crucial for scalable air quality monitoring networks.

This paper also demonstrates the transferability of the developed ML algorithms. Following the initial development and validation using data from a specific measurement site in Athens, Greece, the developed model was successfully applied to a secondary site in Patras, Greece. This transferability confirms the robustness and versatility of the particular ML approach, marking a significant improvement over traditional calibration methods that are often constrained by their context-specific configurations.

## 2. Existing Approaches

In this section, we present and discuss key existing approaches for calibrating similar low-cost sensors. The urgent need to calibrate low-cost sensors for air quality monitoring has driven forward a broad spectrum of research efforts. At first, laboratory calibration efforts were proposed to improve the sensors’ precision. Laboratory calibration under controlled conditions and usually under the presence of only the gas under examination often leads to considerable errors when moving to the field [4,6,7,28,30]. For this reason, several studies have proposed field calibration so that the sensors are exposed to real conditions, which is preferable when designing and deploying calibration methods that rely on historical data. A series of traditional statistical methods, like linear regression (LR), have been tested in the past. Recently, ML algorithms have been employed, which provided better results.

Ariyaratne et al. [31] deployed ten CO-B4 (Alphasense Ltd., Essex, UK) sensors and inspected the calibration performance of the manufacturer’s equations against single linear regression and multiple linear regression (MLR) models. Both models used the sensor voltage signal outputs and temperature and humidity to estimate concentration. Evaluation was performed for 5- and 60-min averages for two weeks using a high-end instrument in Kandy city, Sri Lanka. Using the manufacturer’s equations resulted in a Root Mean Squared Error (RMSE) that ranged between 170 and 338 ppb (at 5 min averages) and between 83 and 249 ppb (at 60 min averages). R^2^ ranged from 0.15 to 0.65 (at 5 min averages) and from 0.29 to 0.9 (at 60 min averages). The authors developed an average MLR model (aggregating 10 sensor-specific MLR models) that yielded a mean absolute error (MAE) ranging from 37.6 to 42.5 ppb (at 5 min averages) and from 44.6 to 127.3 (at 60 min averages). R^2^ ranged from 0.94 to 0.95 at both average times.

Zuidema et al. [12] evaluated multiple CO-B4, NO-B4, NO2-B43F, and OX-B431 sensors (Alphasense Ltd., Essex, UK) for two years in the Puget Sound region of Washington, USA. The authors developed several MLR models that considered the voltage signals of the sensors, the individual sensors’ electronic coefficients, and the measured temperature and humidity. Their work underlined the significance of inter-sensor variations and the influence of temperature and relative humidity on sensor response. Incorporating predictions from low-cost CO sensors into the calibration model for NO_2_ sensors enhanced calibration performance due to their shared traffic-related source. Despite efforts to mitigate ageing effects with auxiliary electrodes, the authors observed sensor drift over time, which varied by gas. For instance, while CO sensors exhibited significant and variable drift, NO_2_ sensors showed more uniform drift.

Cross et al. [17] evaluated CO-B4, NO-B4, NO2-B43F, and OX-B432 sensors (Alphasense Ltd., Essex, UK) at 5 min average over 4.5 months in Boston, MA, USA. They employed high-dimensional model representation methods for calibration and compared them against the equations provided by the manufacturer. Their approach accounted for several parameters besides the sensors’ response, such as environmental factors. The study revealed that the manufacturer’s recommended electrode corrections do not lead to pollutant concentration values of acceptable accuracy for ambient air analysis. The high-dimensional model representation calibration method produced mean absolute errors of 25, 3, 3, and 7 ppb for CO, NO, NO_2_, and O_3_ sensors. The corresponding R^2^ values were 0.88, 0.84, 0.69, and 0.39, respectively. On the contrary, the default calibration method provided by the sensors’ manufacturer yielded R^2^ values of 0.78, 0.21, 0.18, and 0.12, respectively. 

Kim et al. [4] evaluated CO-B4, NO-B4, NO2-B43F, and OX-B432 sensors (Alphasense Ltd., Essex, UK) over two weeks (using hourly averaged values) in the San Francisco Bay area, CA, USA. The study used 50 sensor nodes, mounted on roofs of public buildings. The study used custom-made equations for converting the electronic signal to concentration, considering environmental factors (temperature and relative humidity), and sensors’ cross-sensitivity, as auxiliary variables in the equations. Cross-sensitivity was found for NO_2_ and O_3_ sensors. OX-B431 was sensitive to NO_2_. The NO2-B43F sensor was more sensitive to NO than the sensor manufacturer reported. The same sensor was also sensitive to CO_2_. The study also confirmed that the sensors’ corresponding zero drifts and sensitivities were temperature and age dependent. 

Tryner et al. [32] evaluated the CO-B4, NO2-B43F, and O3-B431 sensors (Alphasense Ltd., Essex, UK), among others, in an indoor colocation setup with reference instrumentation located inside a house in Fort Collins, CO, USA. The study used nine sensor boxes placed in the kitchen of the house. The authors compared the manufacturer’s calibration equations with their linear-regression ones, constructed using the sensor’s electronic response and temperature. Use of the manufacturer’s equations resulted in RMSE of 645, 22, and 21 ppb for CO-B4, NO2-B43F, and O3-B431 sensors, respectively. These errors were reduced to 572, 14, and 7 ppb, respectively, when the authors applied the alternative linear equation under a 7-fold cross-validation.

Casey et al. [33] evaluated the CO-B4 sensor over 3 months in Greeley, CO, USA. For sensor calibration, the authors developed an Artificial Neural Network (ANN) model and simple linear regression using the sensor electrode signals and auxiliary signals from other sensors (CO_2_, O_3_). The authors observed that the ANN model performed similarly at multiple averaging times (ranging from 1 min to 1 h). The collective sensor signals improved the model’s performance, producing an R^2^ of 0.78 and an RMSE of 2 ppb on the test set. 

Han et al. [11] performed 1-year field calibration of CO-B4, NO2-B43F, and OX-B431 sensors at Jiandemen, in Beijing, China. The authors compared single and MLR with a Random Forest (RF) and a Long-short Term Memory (LSTM) model. Linear equations used the sensor’s response in ppb (calculated using the manufacturer’s equations provided by the manufacturer) and ambient temperature and relative humidity. RF and LSTM used the sensor’s electrode responses, ambient temperature, and relative humidity. The authors found the RF and LSTM models were superior to the rest. Also, their analysis suggested that ML can eliminate the effects of temperature and humidity in sensor responses. Finally, they showed that ML can decrease biases at different pollution levels for all three sensors. The post-calibration R^2^ for the CO, NO_2_, and O_3_ sensors was 0.93, 0.84, and 0.77, respectively. The post-calibration RMSE was 166, 11, and 26 ppb, respectively. 

Zimmerman et al. [5] proposed an RF algorithm for calibrating 10–16 sensor packages, including the CO-B4, NO2-B43F, and OX-B431 sensors (Alphasense Ltd., Essex, UK). The measurement site was in Pittsburgh, PA, USA. Training and testing were performed over six months. An RF model was built using the collective sensor package’s net electronic output, ambient temperature, and relative humidity. The authors developed a laboratory-determined simple linear regression model and a multiple linear regression model that used single-sensor net voltage outputs, ambient temperature and/or relative humidity for comparison purposes. The authors concluded that the main strength of the RF approach is that it accounted for pollutant cross-sensitivities and provided better calibration results. For CO, NO_2_, and O_3_, the calibrated ME was 38 ppb (relative error 14%), 3.5 ppb (relative error 29%) and 3.4 ppb (relative error 14%), respectively. All R values exceeded 0.8. 

Kim et al. [28] evaluated the long-term stability and calibration of the NO-B4 and NO2-B43F sensors (Alphasense Ltd., Essex, UK). The sensors were calibrated using an RF model built from 6-month training data in Haerkingen, Switzerland. Next, the sensor units were installed in the city of Zurich for 1 year of continuous operation. Finally, the sensors were co-located again at Haerkingen for another 4 months. The calibration models produced the same errors after 18 months of operation for the NO sensor. However, the NO_2_ sensor’s performance deteriorated (RMSE increased from 3 ppb to 4 ppb after 18 months). The authors also reported that changing sensor installation sites significantly affected the calibration performance.

Using a multiple-stage calibration approach, Cui et al. [6] evaluated the NO2-B43F, OX-B431, and SO2-B4 sensors. During the first two stages, the laboratory calibrated the sensors to correct temperature and humidity influence and verify cross-sensitivity. Linear regression and ML models were developed for this purpose. The authors propose a two-step field calibration. During the first step, sensors are co-located with reference instrumentation to obtain enough data for model building. The second step involves re-calibration after re-locating the sensors. The authors reported significant variability among sensors of the same kind. 

Despite the recent progress in improving the calibration of low-cost sensors, a number of issues remain unresolved. The existing literature on sensor calibration methodologies exhibits several notable limitations. 

It is not clear how sensor variability affects calibration methodologies, thereby potentially impeding the development of robust calibration techniques [5,12]. Some research studies predominantly rely on data from a single training-testing site [32,33]. The latter may limit the generalizability and robustness of the developed calibration methods [8,13,14,21]. The failure to incorporate diverse environmental conditions and sensor behaviors across different locations may lead to calibration strategies that are overly specialized and lack broader applicability. A few studies have proposed the use of electronic signals from sensors [5,31]. However, they often rely on simplistic linear formulas in their calibration approaches. Coupled with the aforementioned limitations regarding sensor variability and single-site testing, this methodology may render these calibration techniques insufficiently robust and incapable of accurately capturing the complex relationships between sensor signals and environmental parameters.

Finally, some works propose leveraging the collective outputs of multiple sensors for calibration purposes [12,27]. However, these approaches frequently rely on a limited number of sensors. The latter may result in calibration methods that inadequately account for the underlying interactions between various environmental parameters, compromising their efficacy in real-world applications.

## 3. Materials and Methods

### 3.1. Research Methodology

**Figure 1:**
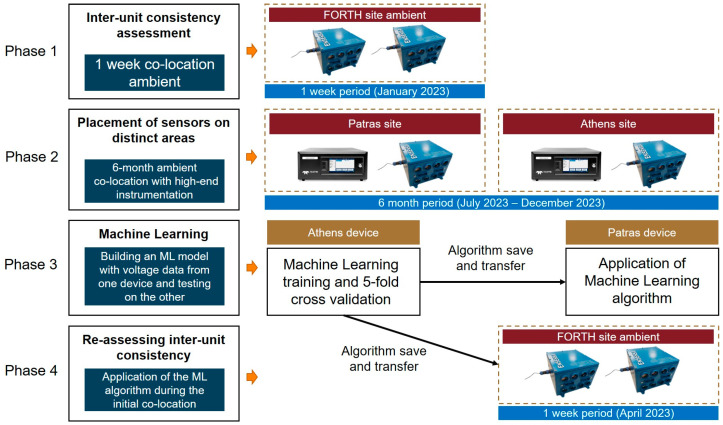
Overview of the experimental phases.

**Figure 2:**
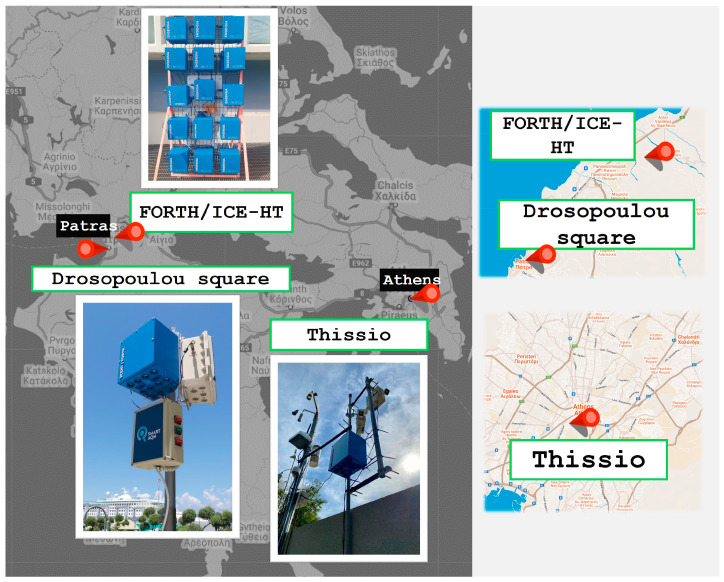
Measurement sites used in this work.

Our methodology is illustrated in Figure 1. Two ENSENSIA (Environment Sensing Appliance) devices [27,29] were used. In the first phase of the work, the devices were placed side-by-side in a suburban location (FORTH/ICE-HT, Patras, Greece) for one week. During the second phase (6 months), one device was placed at the Thissio site in the center of Athens, Greece, and the other at the Drosopoulou square in the center of Patras, Greece (Figure 2). In both these locations, the devices were placed close to high-end instrumentation, which provided the reference concentrations.

**Figure 3:**
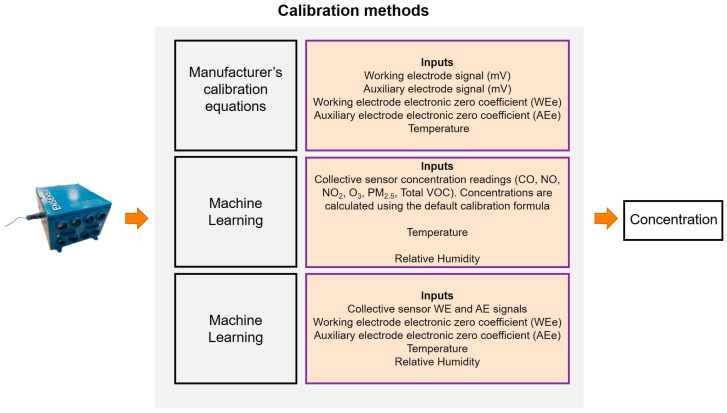
Calibration methods and their inputs.

For the evaluation of the methods, we conducted several analyses (Figure 3). The Athens site was chosen to train and initially validate the ML approaches. Data from the ENSENSIA device placed there and from the reference instrumentation were used to build the ML algorithm that will be applied to both ENSENSIA devices regardless of their location. We then assessed the calibration efficacy of all methods for the Athens device. We adopted a 5-fold cross-validation procedure [28] at the Athens site for the ML methods, which require training data. For the manufacturer’s equation, no training data are necessary. 

Subsequently, we evaluated the performance of the calibration methods at the Patras site (phase 3). Specifically, we quantified the effectiveness of the ML algorithm trained in Athens in calibrating the device at the Patras site, thereby evaluating the generalization capability of the ML model. Hence, measurement data from the Drosopoulou site in Patras were only used for testing. The evaluation of the ML algorithm in Patras was based on concentrations provided by regulatory instrumentation.

Additionally, all methods were employed during phase 4 using the data collected during phase 1 to examine which algorithm minimizes the initial variability of the ENSENSIA devices. During the latter period, no reference instrumentation was present.

### 3.2. ENSENSIA

We have developed a multiple low-cost sensor system for monitoring outdoor and indoor air quality [27,29]. The device’s version that provided the data of the present study contains five electrochemical sensors (CO, NO, NO_2_, O_3_, Total VOCs), one nondispersive infrared CO_2_ sensor, two identical laser sensors for measuring PM_2.5_, and a sensor for measuring temperature and relative humidity. Detailed sensor ranges, models, units, and manufacturers are presented in Table 1.

ENSENSIA uses a Raspberry PI (Model 4B) processing unit to gather the sensor output signals every 10 s. The module transmits data every 2 min (averaged data) to a central server. The present study examined the performance of the CO, NO, NO_2_, and O_3_ sensors. The total VOC and the environmental sensor were used when building the ML models. The PM_2.5_ and CO_2_ sensors were neither studied here nor included in the ML model.

### 3.3. Measurement Sites

#### 3.3.1. Inter-Unit Comparison Site (FORTH/ICE-HT, Patras)

The inter-unit comparison site (Figure 2) is in the suburbs of Patras (38°17′50.604″ latitude, 21°48′31.248″ longitude). The site is around 10 km away from the city centre. Two ENSENSIA devices were placed next to each other in the ICE-HT’s parking lot for one week (20–26 April 2023). The devices were placed 2 m above ground and 30 cm each other. We applied all calibration methods during this setup to investigate their influence on inter-unit consistency. 

#### 3.3.2. The Athens Site

The Thissio Air Quality Monitoring Station (ATMOS-NOA; 37.97° N, 23.72° E, 105 m a.s.l.), operating since 2013, is located at the central premises of the National Observatory of Athens, in the historical center of the city (Figure 2). The station is situated within an area of moderate population density, close to a large pedestrian zone and away from major roads, and it is representative of background pollution in Athens. More details about the site and the air quality characteristics of Athens can be found in [34,35]. Regulatory instrumentation included a Model 49i Ozone Analyzer (Thermo Fisher Scientific, Waltham, MA, USA), a Serinus 40 (Ecotech, Knoxfield, Australia) oxides of nitrogen analyzer, and a G2401 (Picarro, Santa Clara, CA, USA) gas concentration (CO, CO_2_) analyzer. Reference concentrations were available at hourly intervals. We used this site to train and initially evaluate our ML algorithms. In addition, we tested the manufacturer’s calibration equations against the ML-based methods with respect to the high-end instrumentation.

#### 3.3.3. The Patras Site

The third site is situated at the center of Patras, Greece (Figure 2), positioned at a latitude of 38°14′45.976″ and a longitude of 21°44′8.036″. An ENSENSIA device was installed atop a small structure utilized as an official air quality monitoring station by the region of Western Greece. The device was positioned approximately 6 m above ground level and adjacent to the inlets of the reference monitor, which was a 360 (HORIBA, Kyoto, Japan) model capable of measuring CO, SO_2_, NO_x_, and O_3_. Reference concentrations were available at hourly intervals. This site was used for test purposes. More specifically, the ML-based calibration methods, which were trained at the Thissio site, were applied to calibrate the device at this third site.

### 3.4. ENSENSIA Calibration Methods

#### 3.4.1. Manufacturer’s Calibration Equations

Air passes through a permeable membrane to reach the sensor’s surface, where it interacts with the working electrode (WE). This interaction leads to oxidation or reduction reactions, creating an electronic charge balanced by reactions at the counter electrode. These reactions, catalyzed by specific electrode materials, form redox pairs, and the sensor’s current output is directly linked to the measured gas concentration. During operation, the WE maintains a fixed potential while the counter electrode’s potential can vary. A third reference electrode helps to control the WE potential.

The auxiliary electrode (AE) monitors temperature effects and background currents resulting from processes other than the measured gas. A corrected WE current reflecting only the gas’s electrochemical reaction can be obtained by subtracting the AE current from the total WE current. However, the sensing electrode’s potential fluctuates due to continuous electrochemical reactions, leading to long-term deterioration of sensor performance. Understanding and addressing this drift and degradation are crucial for prolonged sensor functionality.

The electrochemical sensors’ manufacturer (Alphasense Ltd., Essex, UK) provides equations to convert the electronic signal from each sensor to concentration values. These equations are provided for each sensor, and they also include the sensor’s WE electronic zero (WE_e_), AE electronic zero (AE_e_), WE zero (WE_0_), AE zero (AE_0_), and sensitivity (S). The corresponding coefficients are unique for each sensor.

The following equations are suggested by the electrochemical sensors’ manufacturer and used in the present study:

$$CO\left(ppb\right)=\frac{\left(WE-WEe\right)-n(T)(AE-AEe)}{S_{CO}}$$

$$NO\left(ppb\right)=\frac{\left(WE-WEe\right)-k(T)(\frac{WE0}{AE0})(AE-AEe)}{S_{NO}}$$

$${NO}_{2}\left(ppb\right)=\frac{\left(WE-WEe\right)-n\left(T\right)\left(AE-AEe\right)}{S_{{NO}_{2}}}$$

$$O_{3}\left(ppb\right)=\frac{\left(WE-{NO}_{2}-WEe\right)-n(T)(AE-AEe)}{S_{O_{3}}}$$

In the above equations, the parameters *k*(*T*) and *n*(*T*) are temperature dependent and are used to account for the influence of temperature on the signals. The sensors’ manufacturer provides the *k*(*T*) and *n*(*T*) values for each sensor.

#### 3.4.2. Machine Learning Algorithm

The Random Forest (RF) algorithm is particularly suitable for calibrating low-cost sensors due to its robustness against overfitting and its ability to handle high-dimensional datasets with numerous input variables, such as those often encountered in sensor data.

We have selected the RF algorithm, due to its effectiveness in similar tasks ([5,27]). This particular method was successfully employed for calibrating the same electrochemical sensors in the works of Zimmerman et al. [5], Han et al. [11], Vajs et al. [14], Bigi et al. [13], and Kim et al. [28]. Moreover, in our recent study [27], we compared RF against other state-of-the-art ML algorithms and the results showed that RF was more suitable for calibrating our low-cost sensors.

RF is an ensemble learning technique in both classification and regression tasks. The algorithm constructs multiple decision trees (termed estimators) that individually process input data and provide predictions autonomously. Each decision tree tries to identify rules correlating observed variables ($X_{i}$) with expected concentrations ($Y_{i}$). These rules are represented as tree splits, wherein a tree may treat data differently according to whether the value is within a certain threshold (e.g., 10 ppb). Such splits are integral to the decision-making processes within the tree, especially in scenarios with multifaceted attributes, where splits might occur based on various attributes.

We adopted RF in different approaches. In the first approach, the inputs to the model are the raw voltage signal outputs of the electrochemical sensors. The electronic coefficients of the sensors were subtracted from the voltage signal readings in the same way as when calibrating using the manufacturer’s equations (e.g., WE_co_ − WE_e,co_ and AE_co_ − AE_e,co_, respectively, in the case of CO sensor).

In the second approach, the concentration readings of the electrochemical sensors were calculated using the manufacturer’s equations, which were subsequently supplied to the ML model. The PM_2.5_ concentration, temperature, and relative humidity were also supplied to the RF algorithm as inputs in both setups.

The operation of the RF algorithm entails the creation of a multitude of decision trees, each trained on a subset of the input data through a process known as bootstrapping. During training, each tree selects a random subset of features (i.e., input variables) to consider when to make splits, further enhancing diversity among the trees within the ensemble. Once trained, the RF algorithm aggregates predictions from all decision trees to produce a final output. This ensemble approach mitigates overfitting and yields robust predictions, making RF well suited for tasks involving complex and high-dimensional data, such as air quality monitoring.

RF was built with some fixed parameters, whereas hyper-parameter selection was performed by the grid search approach. Grid search is a systematic approach to hyper-parameter tuning in ML, which identifies the optimal combination of hyper-parameters for a given model. By exhaustively searching through a specified subset of the hyper-parameter space, it evaluates model performance for each combination.

For measuring the quality of a split, we used the squared error criterion because it helps in reducing the variance within the subsets, leading to more precise and reliable regression models. In addition, the model is built considering all input features when splitting each node. This is ensured by setting the max_features parameter to one. In applications wherein the number of input features is relatively low, setting this parameter to one does not affect overfitting.

The grid search explored different values for n_estimators (10, 100, 300, 700, 1000), max_depth (4, 8, 30, 50, 80), min_samples_split (1, 2, 4), and min_samples_leaf (1, 2, 4). Starting with the number of estimators (n_estimators), the range from 10 to 1000 allows the assessment of how increasing the number of trees impacts model stability and prediction accuracy. This wide range helps identify a sweet spot where additional trees no longer provide significant benefits relative to the increased computational cost. For the maximum depth of a tree (max_depth), the range of 4 to 80 tests both shallow and deep trees. Shallower trees typically generalize better but might be too simplistic for complex patterns, whereas deeper trees can capture more detail but risk overfitting. Exploring this spectrum ensures that the model’s depth is optimally tuned to balance accuracy and generalization.

Regarding min_samples_split and min_samples_leaf, values ranging from 1 to 4 enable evaluation of the model’s sensitivity to splitting nodes and the minimum size of leaf nodes. These parameters affect the model’s ability to learn fine-grained details without excessively memorizing the training data, thereby controlling overfitting. 

After evaluating performance across these combinations, the parameters yielding the best results were selected. The final RF model consists of 1000 decision trees, each limited to a maximum depth of 8 to prevent overfitting. The model operates silently during training and employs the mean squared error criterion to evaluate splits. Additionally, it requires a minimum of 2 samples to split an internal node and to form a leaf node.

### 3.5. Evaluation Metrics

The Mean Error (ME), Fractional Error (FERROR), Fractional Bias (FBIAS), and Normalized Mean Error or relative error (nME) is used to evaluate the predicted values (*P_i_*) against the actual values (*O_i_*). A lower ME indicates that the predictions are closer to average values. FERROR is a metric that quantifies the fractional difference between P_i_ and O_i_. A lower FERROR suggests more accurate predictions. FBIAS measures the fractional difference between *P_i_* and *O_i_*. It computes the ratio of the sum of differences between *P_i_* and *O_i_* to the sum of their averages, all divided by 2 and the total number of data points (*n*). A lower FBIAS indicates more accurate predictions. nME evaluates the average normalized error between *P_i_* and observed values *O_i_*. It computes the absolute difference between *P_i_* and *O_i_*, normalizes it by dividing by *O_i_*, and then calculates the average of these normalized errors over all data points (*n*). In addition, we considered the coefficient of determination (R^2^) for investigating the correlation between the calibrated and the reference concentrations.

The above-mentioned metrics are derived as follows:

$$ME=\frac{\sum_{i=1}^{n}\left|P_{i}-O_{i}\right|}{n}$$

$$nME=\frac{\sum_{i=1}^{n}\left|P_{i}-O_{i}\right|}{\sum_{i=1}^{n}O_{i}}$$

$$FBIAS=\frac{2}{n}\sum_{i=1}^{n}\frac{\left(P_{i}-O_{i}\right)}{\left(P_{i}+O_{i}\right)}$$

$$FERROR=\frac{2}{n}\sum_{i=1}^{n}\frac{\left|P_{i}-O_{i}\right|}{\left(P_{i}+O_{i}\right)}$$

## 4. Results

### 4.1. Carbon Monoxide

#### 4.1.1. Impact of Calibration Methods on Inter-Unit Consistency

All the developed calibration methods were applied to both ENSENSIA devices during their co-location setup for one week at FORTH. The purpose was to examine the effect of these methods on the variability between the two devices when placed next to each other.

**Figure 4:**
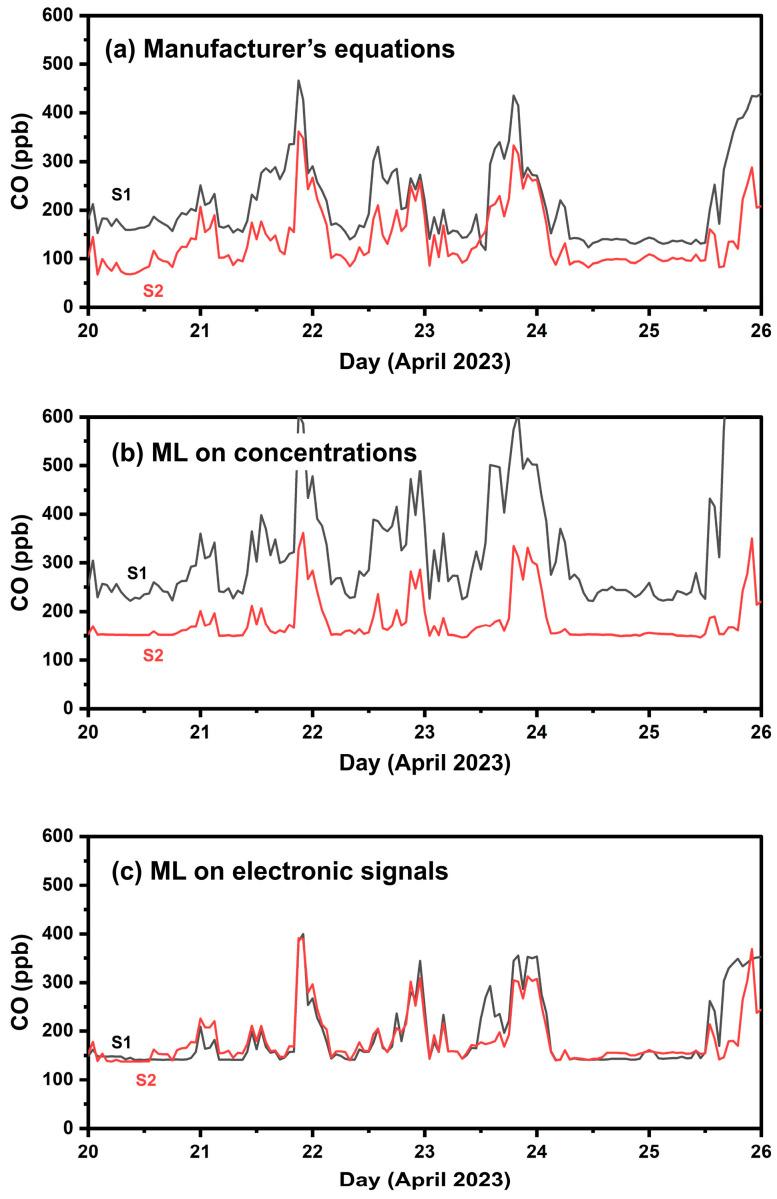
Timeseries of the two CO sensors during the one-week co-location setup (Phase 1) using hourly averaged values. (**a**) Calibration using the manufacturer’s equations, (**b**) calibration applying ML with concentrations as inputs; (**c**) calibration applying ML to the sensors’ electrode response signals.

Figure 4 presents timeseries of the calibrated CO sensors during the one-week colocation setup. Using the manufacturer’s calibration equation resulted in an ME of 73 ppb, an nME of 59% and an R^2^ of 0.65 between the two sensors. The ML based on electrode voltage outputs yields an ME of 20 ppb, nME of 11% and 0.72 R^2^. The application of the ML algorithm using concentrations based on the manufacturer’s equations as inputs exhibits an ME of 158 ppb, an nME of 87% and R^2^ of 0.5.

The ML based on electrode voltage outputs produces results with significantly less variability between the measured concentrations by the two sensors. However, significant variability was still observed on the last day (Figure 4). The observed variability between the two sensors over a single day, while showing consistent performance on other days, suggests that the anomaly might be due to an artifact in the sensor responses. Possible causes include temporary dust or a bug obstructing the sensor hole, which could have impacted the readings. Additionally, this variability might stem from the momentary poor performance of the ML method used to interpret the data. Given these possibilities, further examination is necessary to identify the exact cause of the discrepancy and ensure the reliability of the sensor data.

#### 4.1.2. Calibration Performance in Athens

**Figure 5:**
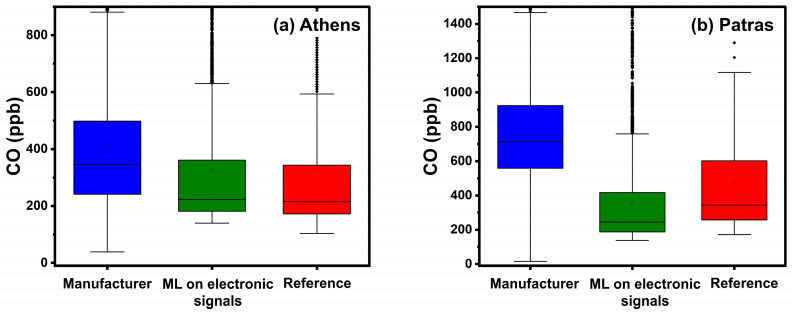
Boxplots for hourly averaged CO concentrations in Athens and Patras. The box represents the interquartile range and the line inside indicates the median. Whiskers extend to 1.5 times the interquartile range from the quartiles, with outliers depicted as points beyond this range.

The performance of each calibration method in Athens was investigated under a 5-fold cross-validation procedure. It should be noted that data from the Athens site were used for training and validating the ML algorithms. The Patras site was used solely for testing purposes and the collected data were not used for extra training. Metric-based results are shown in Table 2. Boxplots of hourly averaged concentrations are presented in Figure 5. Figure S1 shows scatter plots between the multiple calibration methods and the reference hourly averaged CO concentrations. The manufacturer’s calibration equations exhibited an ME of 135 ppb, nME of 55%, and R^2^ of 0.69. This method overestimated the actual CO concentration (FBIAS = 0.44) as observed in Figure S1a. 

Calibration using ML based on sensor electronic signals showed the lowest ME (61 ppb), nME (16%), and the highest R^2^ (0.82). ML using concentration as inputs resulted in an ME of 68 ppb and R^2^ of 0.81. Both ML methods had similar FBIAS (0.05) and nME (16% and 19%, respectively) scores. The boxplots of Figure 5 illustrate the effectiveness of the ML methods. ML using concentrations as inputs underestimated concentrations above 1500 ppb, whilst ML based on sensor electronic signals overestimated concentrations above 1250 ppb (Figure S1c,e). 

#### 4.1.3. Calibration Performance in Patras

The calibration performance in Patras was inspected by applying the ML algorithms that were derived from the ENSENSIA device in Athens to the ENSENSIA device in Patras. Metric-based results at the Patras site are presented in Table 3.

**Figure 6:**
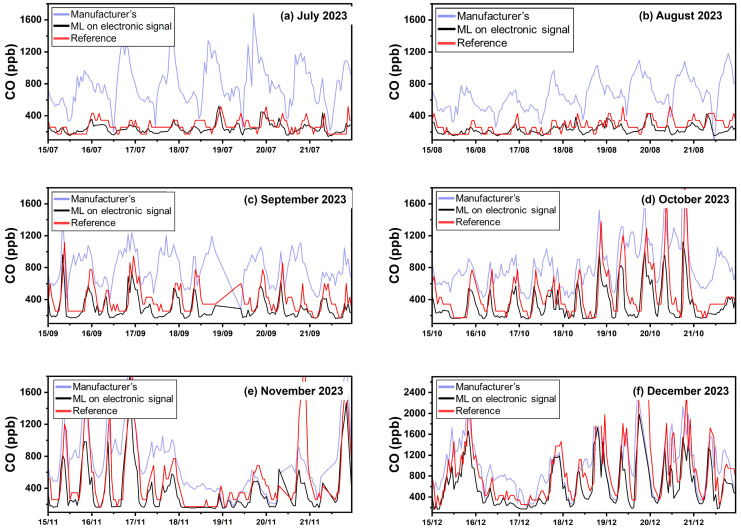
Timeseries for CO in Patras using hourly averaged concentrations.

The manufacturer’s calibration equations exhibited an ME of 353 ppb, nME of 115%, and R^2^ of 0.38. This method significantly overestimates the actual CO concentration (FBIAS = 1.09). The performance of the manufacturer’s equations on this site is significantly different than in Athens. During July and August 2023, the manufacturer’s equation overestimates the CO concentrations (Figure 6, Figures S2 and S4). This behavior can be attributed to high temperature, which interferes with the sensors [21,23,24].

Calibrating using ML based on sensor electronic signals gave an ME of 162 ppb, nME of 31% and an R^2^ of 0.64. There is a slight underestimation of the actual CO concentration (FBIAS = −0.18). The ML using concentration inputs (as calculated using the manufacturer’s equations) produced a slightly lower ME (146 ppb) and a slowly higher R^2^ (0.63). Both ML methods showed comparable FERROR (0.36 and 0.3, respectively) and nME (31% and 32%, respectively). Both ML methods minimized the effect of temperature in the Patras site during the summer period (Figure 6).

### 4.2. Ozone

#### 4.2.1. Impact of Calibration Methods on Inter-Unit Consistency

**Figure 7:**
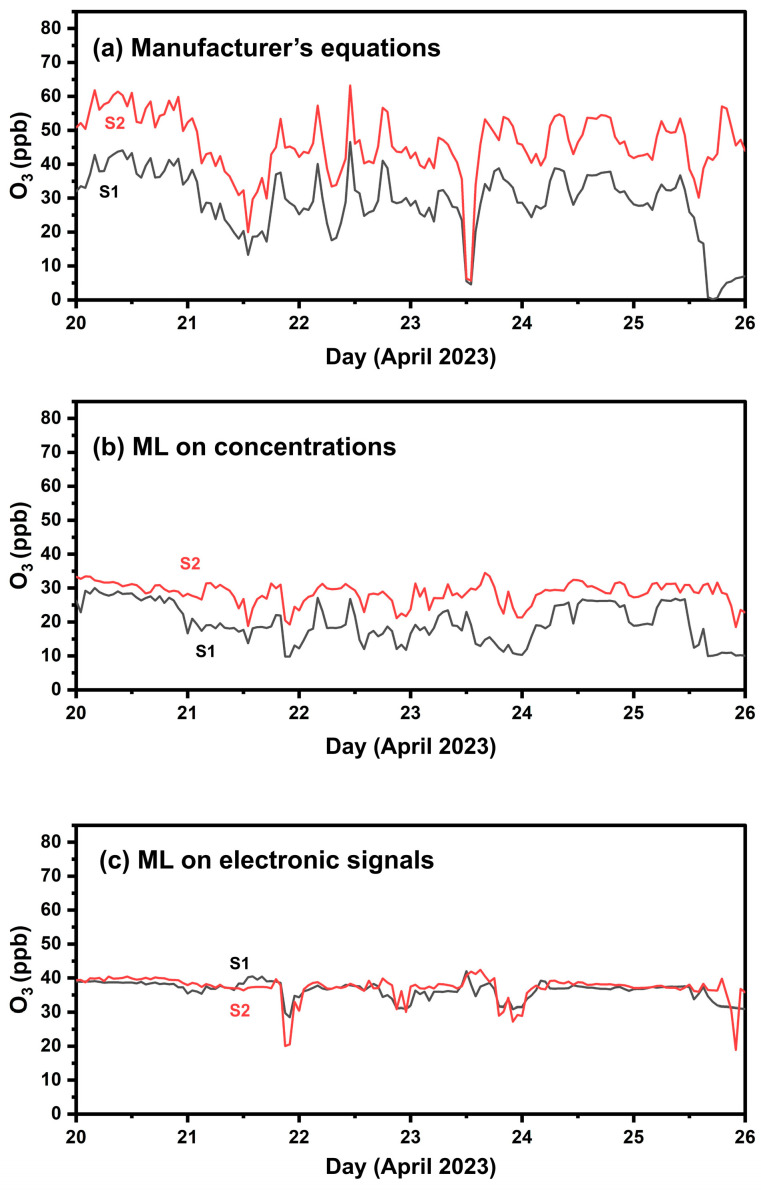
Timeseries of the two O_3_ sensors during the one-week co-location setup (phase 1) using hourly averaged values. (**a**) Calibration using the manufacturer’s equations, (**b**) calibration applying ML with concentrations as inputs; (**c**) calibration applying ML to the sensors’ electrode response signals.

During the one-week colocation period at FORTH, the manufacturer’s equations resulted in an ME of 17 ppb, nME of 37% and R^2^ 0.48 for the comparison between the two devices. Employing ML techniques based on sensors’ electrode outputs resulted in a reduced ME of 5 ppb, a reduced nME (28%) and a slightly better R^2^ (0.5). Applying the ML algorithm with manufacturer’s concentrations as inputs yielded an ME of 12 ppb, nME 31% and R^2^ 0.41. Figure 7 presents timeseries of the measured O_3_ by the two sensors during the one-week colocation setup at FORTH.

#### 4.2.2. Calibration Performance in Athens

**Figure 8:**
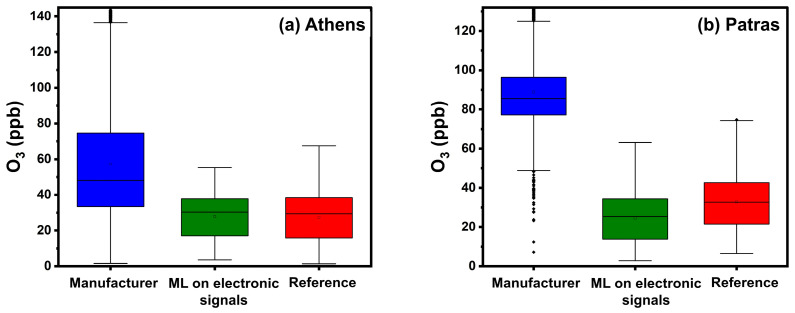
Boxplots for hourly averaged O_3_ concentrations in Athens and Patras. The box represents the interquartile range and the line inside indicates the median. Whiskers extend to 1.5 times the interquartile range from the quartiles, with outliers depicted as points beyond this range.

Table 4 summarizes the calibration results in Athens. When correcting values with the manufacturer’s equations, the ME was 31 ppb (nME 75%), and R^2^ was 0.64. This approach overestimated the actual ozone concentration, as indicated by the fractional bias of 267%. The latter can also be observed in Figure S6, particularly for concentrations higher than 40 ppb. Significant overestimation, especially above 40 ppb O_3_, was also observed in the box plots (Figure 8).

Calibration employing RF based on sensor electronic signals had the best performance, with a notably reduced ME (5.5 ppb) and nME (25%), followed by the highest R^2^ (0.78). 

Applying RF to concentration inputs calculated via the manufacturer’s equations yielded a slightly higher ME of 5.6 ppb, 25% nME, and a higher R^2^ (0.72). The application of ML based on sensor electronic signals resulted in the lowest FBIAS of 22%, indicating a significant improvement.

Both ML approaches seem to work well in the observed concentration ranges, reducing the overestimation that was observed for concentrations above 40 ppb of O_3_ (Figure S6a,c,e). 

#### 4.2.3. Calibration Performance in Patras

**Figure 9:**
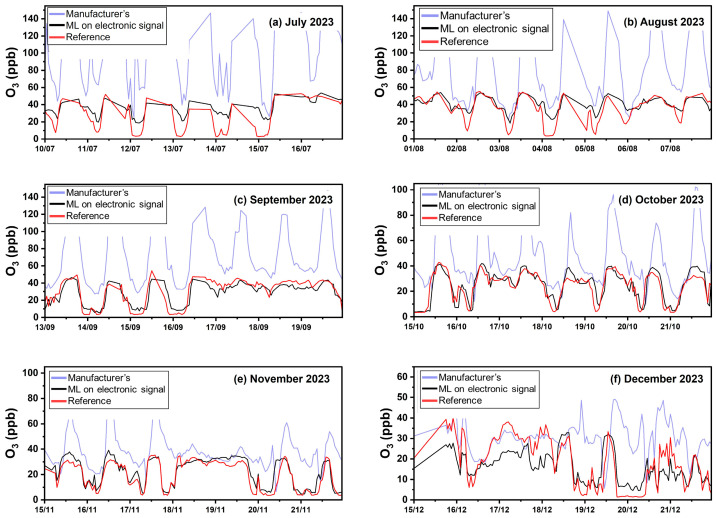
Timeseries for O_3_ in Patras using hourly averaged concentrations.

All results from the Patras site are presented in Table 5. Figure 9 presents timeseries at the third site using hourly averaged concentrations.

Calibration using the manufacturer’s equations resulted in a significant overestimation (FBIAS = 222%, Figure S6b) and gave an ME of 56 ppb, an nME of 222% and an R^2^ of 0.48. During most months, the manufacturer’s equation overestimates the O_3_ concentrations. Specifically during July, August, and September, the overestimation errors can be attributed to higher temperature concentrations, which influences the sensors’ responses [5].

Calibration using ML based on concentration inputs from multiple sensors yielded the lowest ME (9 ppb) and nME (26%). FERROR and FBIAS were 0.28 and 0.1, respectively. Calibration using ML based on sensor voltage signal outputs performed similarly (ME of 9 ppb, R^2^ 0.58, FBIAS 0.2, FERROR 0.4, and nME of 33%).

ML methods eliminated the large biases of the manufacturer’s calibration during the entire period (Figure 9, Figures S9 and S10).

### 4.3. Nitrogen Dioxide

#### 4.3.1. Impact of Calibration Methods on Inter-Unit Consistency

**Figure 10:**
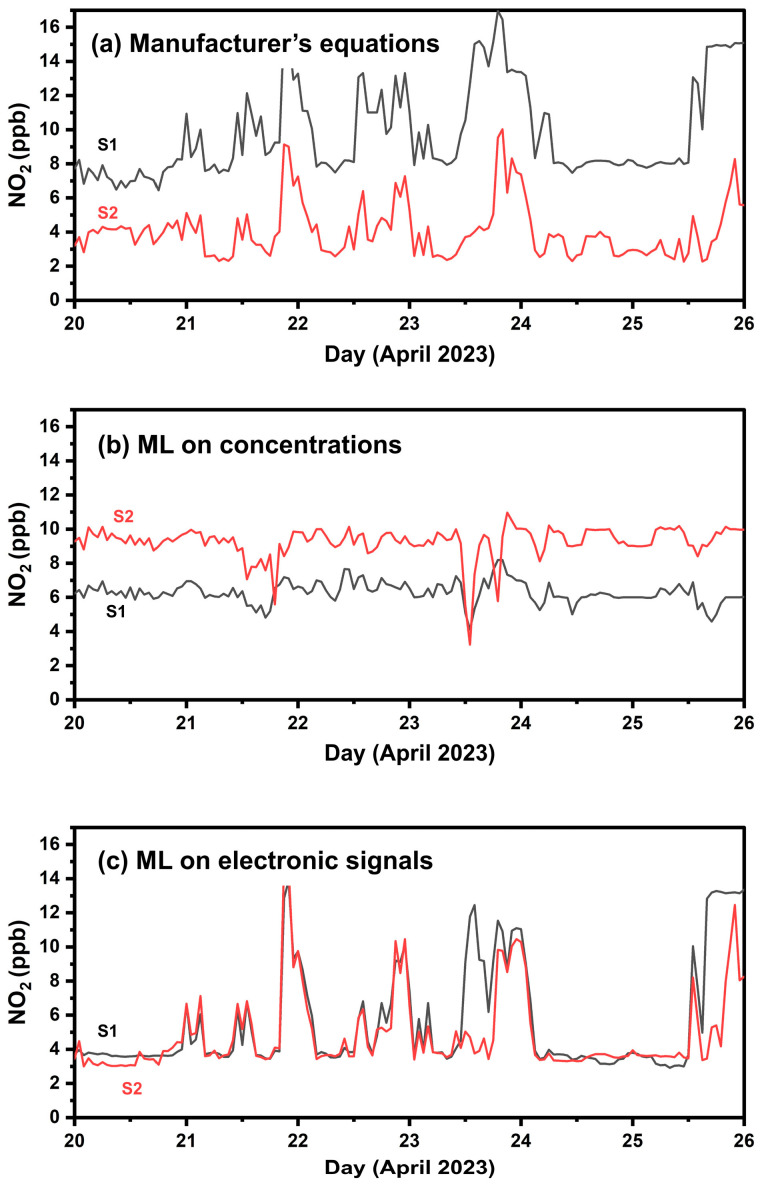
Timeseries of the two NO_2_ sensors during the one-week co-location setup (phase 1) using hourly averaged values. (**a**) Calibration using the manufacturer’s equations, (**b**) calibration applying ML with concentrations as inputs; (**c**) calibration applying ML to the sensors’ electrode response signals.

Figure 10 presents timeseries of measured NO_2_ by the two ENSENSIAs during the one-week colocation setup at FORTH. The two sensors had an ME of 3 ppb, 33% nME, and an R^2^ of 0.25. However, the implementation of ML techniques, specifically based on electrode voltage outputs, presented a notable reduction in sensor variability. ML using electrode signals as inputs exhibited a reduced ME of 1 ppb (nME = 21%) and a substantially higher R^2^ equal to 0.7.

#### 4.3.2. Calibration Performance in Athens

**Figure 11:**
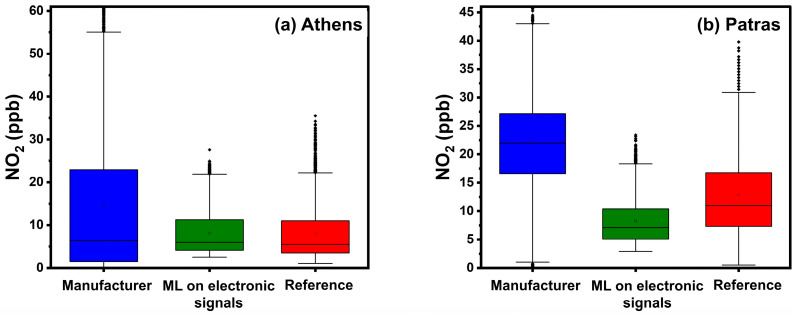
Boxplots for hourly averaged NO_2_ concentrations in Athens and Patras. The box represents the interquartile range and the line inside indicates the median. Whiskers extend to 1.5 times the interquartile range from the quartiles, with outliers depicted as points beyond this range.

Table 6 and Figure 11 summarize the results in Athens. The manufacturer’s equations exhibited an ME of 15 ppb, nME of 326%, and an R^2^ 0.1. This approach significantly overestimates the actual NO_2_ concentration, as indicated by the FBIAS of 2.55 and Figure S11. Calibration employing ML based on sensor electronic signals had the best performance, with a reduced ME of 2 ppb, nME 37% and the highest R^2^ of 0.7. Both ML methods had low FBIAS (0.2).

#### 4.3.3. Calibration Performance in Patras

**Figure 12:**
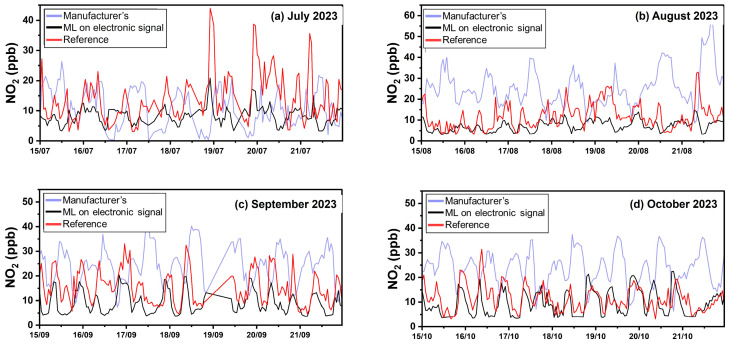
Timeseries for NO_2_ in Patras using hourly averaged concentrations.

Results from the third site are presented in Table 7 and Figure 11. Figure S11 illustrates scatter plots between the multiple calibration methods and the reference NO_2_ concentrations at the second and third sites. Figure 12 presents NO_2_ timeseries in Patras using hourly averaged concentrations and Figure 11 the corresponding boxplots.

Calibration using the manufacturer’s equation was not able to capture the trends in NO_2_ (Figure 12) and produced large bias errors (FBIAS = 1.65). The manufacturer’s calibration equations lead to overestimation errors as observed during August, July, and October (Figure 12 and Figures S12–S14). ML calibration using sensor voltage signal outputs produced less biased results. Although errors during summertime are expected due to high temperatures, the observed errors during October indicate poor performance of the manufacturer’s calibration equations during the entire period. Calibration using ML based on sensor voltage signal outputs yielded an ME of 6 ppb and nME of 42%, followed by an improved R^2^ 0.31. Calibration using ML based on concentration inputs yielded the lowest ME (5 ppb).

### 4.4. Nitrogen Oxide

#### 4.4.1. Impact of Calibration Methods on Inter-Unit Consistency

**Figure 13:**
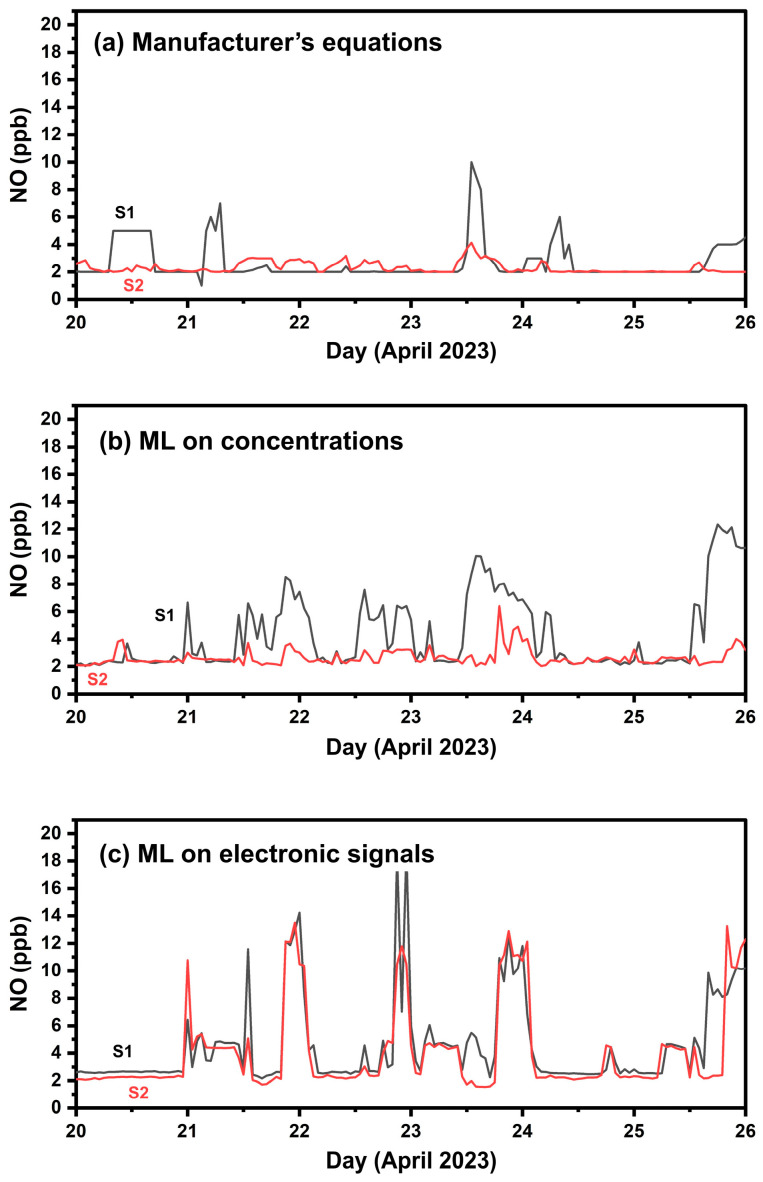
Timeseries of the two NO sensors during the one-week co-location setup (phase 1) using hourly averaged values. (**a**) Calibration using the manufacturer’s equations, (**b**) calibration applying ML with concentrations as inputs; (**c**) calibration applying ML to the sensors’ electrode response signals.

Figure 13 presents measurements of the two NO sensors during the one-week colocation setup at FORTH. RF based on electrode voltage outputs resulted in the lowest ME of 1 ppb, nME of 33% and an R^2^ of 0.7.

#### 4.4.2. Calibration Performance in Athens

**Figure 14:**
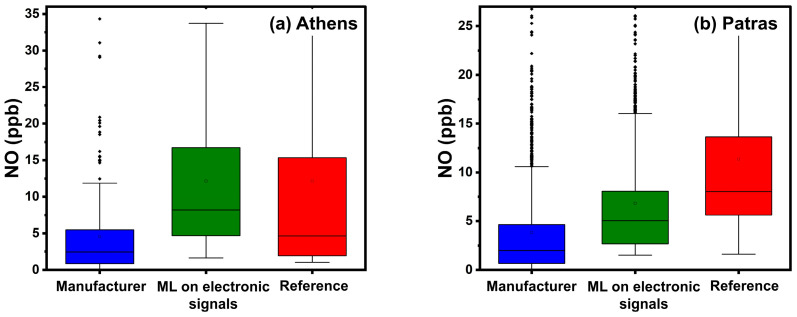
Boxplots for hourly averaged NO concentrations in Athens and Patras. The box represents the interquartile range and the line inside indicates the median. Whiskers extend to 1.5 times the interquartile range from the quartiles, with outliers depicted as points beyond this range.

Table 8 summarizes the results in Athens. The manufacturer’s calibration equations had an ME of 15 ppb (nME = 500%), and an R^2^ of 0.38. The boxplot of Figure 14 indicates poor performance of the manufacturer’s calibration equations in measuring concentrations above 5 ppb of NO. The two ML methods performed similarly, yielding an ME of 6 ppb (nME 100–120%), and R^2^ of 0.69.

#### 4.4.3. Calibration Performance in Patras

All results from Patras are presented in Table 9. Supplementary Figure S4 illustrates scatter plots between the multiple calibration methods and the reference NO concentrations in Athens and Patras. Figure 12 presents timeseries at the Patras site using hourly averaged concentrations.

**Figure 15:**
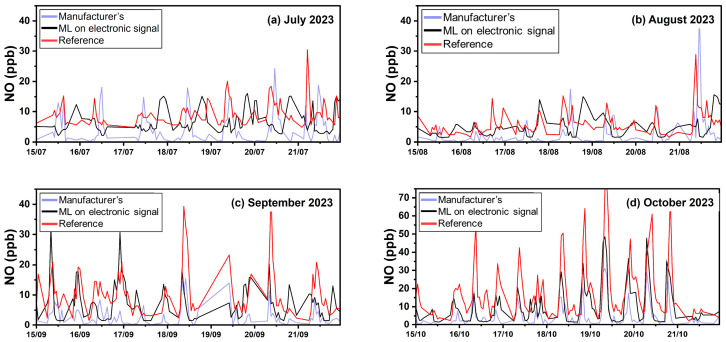
Timeseries for NO in Patras using hourly averaged concentrations.

The manufacturer’s calibration equations yielded an ME of 8 ppb (nME 80%, R^2^ 0.22). For most of the period, this method underestimates the NO concentration (Figure 15), producing an FBIAS of −0.6. At both sites, the manufacturer’s equation performs poorly in measuring NO concentrations above 5 ppb, as confirmed by the boxplots of Figure 14 and by the scatter plots of Figure S15. Calibration using ML based on sensor electrode signals produced an ME of 5 ppb (nME 60%, R^2^ 0.35). However, the latter method also missed some important NO spikes, ranging from 20 to 50 ppb (Figure 15, October 2023). 

The latter behavior is confirmed by the boxplot for the third site (Figure 14). For the summer period, both ML methods performed better.

### 4.5. Importance of the Input Features to the RF Algorithm

We also evaluated the importance of each input feature of the RF algorithm to the outcome, according to the gain method. This technique measures each feature’s average contribution to the augmentation in predictive accuracy when randomly added in the mix during the training of the decision trees in the RF model. This contribution reveals how considerably a feature can influence the predicted output by reducing the impurity or randomness in the model. So, features making an average large, positive contributions are given a higher importance percentage.

For CO, the most important predictor was the CO sensor signal (82.5%), followed by the VOC sensor signal (11.5%). The third most important predictor was the NO sensor (2.5%). Temperature and relative humidity were not considered essential predictors (0.7% and 0.4%, respectively). 

For O_3_, the O_3_ sensor was the strongest predictor (40%), followed by the NO sensor (33%). The CO sensor also had an effect (8.4%). Temperature and relative humidity had effects of 2.4% and 3.4%, respectively.

For NO_2_, the corresponding sensor signals had the highest effect (61%). The VOC sensor was also a significant predictor (14%), followed by the CO sensor (8%). Again, temperature and relative humidity had weak effects (1.7% and 2.3%, respectively).

Finally, the most important predictor for NO was the VOC sensor (47.5%), followed by the NO sensor (29.5%). The CO sensor had an effect of 5%. Temperature and relative humidity affected the ML output less (4.5% and 1.5%, respectively).

The RF algorithm indicated that temperature and relative humidity factors possessed low significance when predicting the concentration of CO, O_3_, NO_2_, and NO. This might seem contradictory, since existing literature highlights that these factors can impact sensor performance. However, it is important to underline that the RF feature importance measurement does not always reflect the absolute truth. Moreover, the effects of these factors are quantified in comparison to the rest of the algorithm’s input features. Finally, temperature and humidity are expected to influence the sensor’s performance particularly during the summer period, which is represented by approximately one third of the data. Hence, the importance of these features is inconsistent across the entire period.

In conclusion, while RF features provide an insightful approach to understanding the relevance of specific factors within an ML model, it should not be the sole method of determining the importance of features in a real-world context. These findings offer an essential dimension of the complex reality behind air quality predictions and are valuable guides for focusing future research efforts.

### 4.6. Seasonal Variation in Calibration Performance

Tables S1–S4 present the seasonal variation in R^2^ and nME for CO, NO, NO_2_, and O_3_ across the two measurement sites (Athens and Patras) for all calibration methods. 

The performance metrics associated with the ML calibration approach based on electronic signals showed variations across different seasons, notably between the summer and other seasons. 

In Athens, for CO, the measurements showed the highest nME during the summer season at 0.21, while the lowest nME was observed during the winter season at 0.14. The R^2^ score was the lowest during the summer at 0.75 and achieved its highest value in the winter season at 0.85. Similar trends for O_3_ were observed in the same measurement site. The nME was the highest in the summer season at 0.22, significantly higher than the winter season (0.19). The R^2^ value was also lower in summer at 0.7 compared to winter, where it achieved the highest value at 0.81. NO_2_ followed the same trend in Athens. The summer season saw the highest nME of 0.45. Meanwhile, the winter season brought the lowest nME at 0.25.

Regarding R^2^, summer demonstrated a lower value at 0.64 while winter had the highest value of 0.79. For NO, summer exhibited the highest nME at 1.5, while winter had the lowest nME at 1.1. Similar to the other pollutants, R^2^ was lower in summer at 0.54 and highest in winter at 0.77.

Shifting to Patras, similar patterns were observed, though with some minor differences. For CO, the summer period demonstrated the highest nME of 0.36 and the lowest during winter at 0.14. R^2^ values were the lowest in the summer at 0.6 and highest in the winter at 0.72. Concerning O_3_, the highest nME occurred in the summer at 0.39 and the lowest nME during the winter at 0.19. The lowest value of R^2^ was recorded in summer at 0.52 and the highest value was recorded in winter at 0.69. In the case of NO_2_, nME values were the highest during the summer at 0.52 and lowest in the winter at 0.38. Patras saw the lowest accuracy in R^2^ values during the summer at 0.2 and the highest in winter at 0.45. Lastly, for NO, Patras experienced the highest nME during summer at 0.8 and the lowest during winter at 0.3. Like the other pollutants, R^2^ was the lowest in summer at 0.35 and the highest in winter at 0.5. In both Athens and Patras, regardless of the pollutant, the model’s performance (in terms of both the nME and R^2^ parameters) consistently deteriorates during the summer season and improves in the winter. Again, similar observations could be made for the rest of the calibration approaches with notably stronger variations.

These observations imply that the summer season presents more challenges in predicting pollutant levels, potentially due to complex interactions of meteorological factors. Conversely, the winter period yields better predictive model performance, indicating comparatively stable atmospheric conditions and lower pollutant emissions.

### 4.7. Comparison against Existing Approaches

We compared the results with key literature findings. The results are presented in Table S5. For the measurement of CO, our study’s performance in Athens shows an R^2^ value of 0.82, while for Patras, it is 0.63. Other studies present a range from 0.54–0.95. The latter suggests that our method’s performance lies in the middle of this spectrum. Regarding ME, our results span from 61 ppb to 162 ppb and in the broader scope, they range from 24.8 ppb (Cross et al. [17]) to 289 ppb (Papaconstantinou et al. [25]).

Analyzing NO, the studies present an R^2^ range between 0.35 (our study in Patras) and 0.97 (Zuidema et al. [12]). Our approach in Athens achieved an R^2^ of 0.69, closer to the high end of the spectrum. Regarding ME, our results for Athens and Patras were 6 and 5 ppb, respectively, displaying values entirely within the range of other studies (2.1 ppb to 4 ppb).

Considering NO_2_, our R^2^ values (0.7 for Athens and 0.31 for Patras) stand at both ends of the spectrum in comparison to other papers, ranging from 0.54 (Papaconstantinou et al. [25]) to 0.89 (Vajs et al. [14]). Our ME results (2 ppb in Athens and 6 ppb in Patras) are within the scope of other studies (3 ppb to 11 ppb).

Lastly, for the study of O_3_, our R^2^ of 0.78 and 0.58 (for Athens and Patras, respectively) falls within the observed range of 0.05–0.81. Our ME values (5.5 ppb for Athens and ten ppb for Patras) also lie within the scope of 3.4 ppb (Zimmerman et al. [5]) to 48 ppb (Papaconstantinou et al. [25]).

The present results collectively are consistent with existing peer research. Although our experiment was conducted over nine months, the other studies had varying periods, ranging from a minimum of 4 months (Cross et al., Zimmerman et al., Bigi et al. [5,13,17]) to a maximum of 2 years (Zuidema et al. [12]). This variance could significantly impact the consistency and reliability of the measured parameters as the longer the period, the more robust the environmental data collected, potentially leading to more precise results.

Notably, we trained and tested our algorithms at two sites, Athens and Patras, rather than the more common approach of conducting the training and evaluation on the same site with the same sensor. We merely divided the data collection period into training and testing segments. This method provided us with an additional dimension of validation, as it tests the model’s applicability and transferability across different sites. Despite the possibly more challenging scenario, the performance of RF remains within the ranges presented by other studies. This demonstrates its suitability and capacity to perform well regardless of geographical variances, enhancing its potential for real-world application.

It is apparent that discrepancies among the results of diverse studies, including ours, may be attributed to several factors. These factors underscore the inherent complexity of environmental data collection and analysis and the challenges in producing compatible comparative evaluations.

Firstly, the experiments were conducted in different geographical locations, each with unique environmental conditions and ambient pollutant levels. This geographical variation can lead to different results, even when the same experimental parameters are used.

Concentration exposure, or the amount of pollutants to which the sensors are exposed, also plays an important role. Even subtle changes can affect the sensors’ performances, consequently influencing both the calibration success and the precision of detection results.

Finally, the calibration methodologies employed by each study may vary quite significantly. Various forms of LR, ML models like RF, LSTM, and heterogeneous data modelling regression are used. Each of these methods has strengths and weaknesses, and their suitability may vary depending on the specific research context.

In conclusion, while the calibration performance of this study in the context of these considerations was comparable to related works, it is crucial to recognize that comparisons should account for these inherent complexities. Therefore, exact alignment in results should not be expected but rather comparable performance ranges, as reflected in our data.

## 5. Discussion

We proposed a general ML method based on the RF model for calibrating electrochemical low-cost sensors for use in urban areas. Two urban measurement sites with high-end instrumentation were used for training and testing over eight months. A third site (suburban) was selected to evaluate the effect of the calibration methods on inter-unit variability among the deployed sensors.

The ML approach was superior to the manufacturer’s calibration equations at both sites. Inspection of the inter-unit variability during the ENSENSIA colocation period showed that the manufacturer’s equations introduced significant variability between the CO, O_3_, and NO_2_ sensors (73 ppb ME for the CO sensors, 17 ppb ME for the O_3_ sensors, 3 ppb ME for the NO_2_ sensors). Calibration using ML with the collective sensor voltage responses reduced the observed variability (20 ppb ME for the CO sensors, 5 ppb ME for the O_3_ sensors, 1 ppb ME for the NO_2_ sensors). Calibrating using ML with concentration inputs introduced variability between the sensors during ambient colocation at FORTH, although performed similarly to calibration using electrode outputs in Athens and Patras (with reference to the regulatory instrumentation). This behaviour can be attributed to the concentration profile of the FORTH site, a suburban site with cleaner air compared to the rest of the measurement sites.

For the CO sensor, the ME and relative error (nME) of the RF approach based on the sensor’s electrode outputs in Athens (training and validation site) were 61 ppb and 16% (R^2^ = 0.82). In Patras (test site), the ME was 162 ppb (31% relative error, 0.64 R^2^). For the O_3_ sensor, the ME and nME in Athens were 5.5 ppb and 25% (R^2^ = 0.78). In Patras, the ME was 10 ppb (33% relative error, 0.58 R^2^). For the NO_2_ sensor, the ME and nME in Athens were 2.4 ppb and 37% (R^2^ = 0.7). In Patras, the ME was 5.6 ppb (42% relative error, 0.31 R^2^). For the NO sensor, the ME and nME in Athens were 6 ppb and 120% (R^2^ = 0.69). At the Patras’ site, the ME was 5 ppb (60% relative error, 0.35 R^2^). Notably, RF calibration for the NO sensor performed better in Patras.

We conclude that ML based on concentration inputs has less generalization capabilities. On the contrary, ML based on the sensor’s electrode responses showed better transferability because it minimized the inter-unit variability. Moreover, the latter ML method considers the electronic coefficients accompanying each sensor and uses the collective sensor responses for estimating individual pollutants. Hence, it is more robust to slight variability in the WE and AE signals present in such sensors.

Cross-sensitivity is a significant characteristic of low-cost sensors. The study demonstrates that building ML calibration models that use multiple sensor outputs can take advantage of the cross-sensitivity and better estimate the pollutant concentrations.

A limitation of the study is the period of the data utilized for calibration. The proposed methods were tested over six months, not an entire year. There is a potential that the uncovered findings might not present a holistic view of sensor performance, as variations in environmental factors across different seasons can influence sensor responses. This could affect the performance of the applied methods to varying degrees throughout the year. In addition, the effects of ageing on the sensors have not been studied. As the sensor units age, their performance characteristics will likely change over time, potentially impacting the observed calibration results. Therefore, excluding this factor limits the generalizability of the conclusions drawn. Future research is necessary to investigate this phenomenon more thoroughly by incorporating longitudinal studies that take into account data over prolonged periods covering the lifecycle of the sensor.

Next, the scope of the experimental sites was geographically limited, with only two urban and one suburban site involved in the study. More locations would offer varied environmental conditions, which would help comprehensively evaluate the accuracy and transferability of the calibration methods.

Lastly, our analysis revealed that ML based on the sensor’s electrode signals successfully minimized inter-unit variability at the suburban site (FORTH); however, this aspect was not assessed for urban sites. Thus, future expansion of this research would ideally incorporate an examination of inter-unit consistency of the electrochemical sensors at urban sites for a more rounded understanding of the suitability of ML calibration methods in different geographic scenarios.

## 6. Conclusions

This study proposed a promising pathway to improve the calibration efficiency of low-cost electrochemical air quality sensors for CO, NO, NO_2_, and O_3_. Applying the RF ML model directly to the sensors’ electrode response signals reduces inter-unit variability. It improves accuracy in predicting pollutant concentrations in multiple test sites across Greece. Applications of the RF model using voltage signals directly from sensors performed better than using manufacturer-provided correction equations. The study also demonstrates the effectiveness of exploiting sensor cross-sensitivity to enhance calibration performance. Further studies are necessary to validate the findings in different settings and for other gases.

## Figures and Tables

**Table 1:** Description of the low-cost sensors used in ENSENSIA.

Target Pollutant	Sensor Model	Range	Manufacturer
Ozone (O_3_)	OX-B431	0–200 ppb	Alphasense
Nitrogen Dioxide (NO_2_)	NO2-B43F	0–200 ppb	Alphasense
Nitric Oxide (NO)	NO-B4	0–200 ppb	Alphasense
Carbon Monoxide (CO)	CO-B4	0–2000 ppb	Alphasense
Total VOCs (tVOCs)	VOC-B4	0–10,000 ppb	Alphasense
Carbon Dioxide (CO_2_)	COZIR-AH	0–10,000 ppm	Gas Sensing Solutions
Fine Particle Matter (PM_2.5_)	PMS5003	0–500 μg m^−3^	Plantower
Temperature	BME680	−40–85 °C	Bosch Sensortec
Relative Humidity	BME680	0–100%	Bosch Sensortec

**Table 2:** CO performance metrics in Athens.

Method	ME (ppb)	FERROR	nME	FBIAS	R^2^
Manufacturer’s equations	135	0.39	0.55	0.44	0.69
ML on concentrations	68	0.18	0.19	0.05	0.81
ML on electronic signals	61	0.15	0.16	0.05	0.82

**Table 3:** CO performance metrics in Patras.

Method	ME (ppb)	FERROR	nME	FBIAS	R^2^
Manufacturer’s equations	353	0.63	1.15	1.09	0.38
ML for concentrations	146	0.3	0.32	0.05	0.63
ML for electronic signals	162	0.36	0.31	−0.18	0.64

**Table 4:** O_3_ performance metrics in Athens.

Method	ME (ppb)	FERROR	nME	FBIAS	R^2^
Manufacturer’s equations	31	0.75	0.75	2.67	0.64
ML for concentrations	5.6	0.28	0.28	0.69	0.72
ML for electronic signals	5.5	0.25	0.25	0.22	0.78

**Table 5:** O_3_ performance metrics in Patras.

Method	ME (ppb)	FERROR	nME	FBIAS	R^2^
Manufacturer’s equations	56	0.96	2.22	2.22	0.48
ML for concentrations	9	0.28	0.26	0.1	0.57
ML for electronic signals	10	0.4	0.33	0.2	0.58

**Table 6:** NO_2_ performance metrics in Athens.

Method	ME (ppb)	FERROR	nME	FBIAS	R^2^
Manufacturer’s equations	15	1.22	3.26	2.53	0.1
ML on concentrations	2	0.32	0.38	0.2	0.66
ML on electronic signals	2	0.31	0.37	0.2	0.7

**Table 7:** NO_2_ performance metrics in Patras.

Method	ME (ppb)	FERROR	nME	FBIAS	R^2^
Manufacturer’s equations	13	0.75	1.8	1.65	0.1
ML on concentrations	5	0.46	0.45	0	0.23
ML on electronic signals	6	0.5	0.42	0.1	0.31

**Table 8:** NO performance metrics in Athens.

Method	ME (ppb)	FERROR	nME	FBIAS	R^2^
Manufacturer’s equations	15	1	5	5	0.38
ML on concentrations	6	0.9	1	0.9	0.69
ML on electronic signals	6	0.6	1.2	1	0.69

**Table 9:** NO performance metrics in Patras.

Method	ME (ppb)	FERROR	nME	FBIAS	R^2^
Manufacturer’s equations	8	1.2	0.8	−0.6	0.22
ML on concentrations	7	0.7	0.7	0	0.15
ML on electronic signals	5	0.6	0.6	0	0.35

## Data Availability

The study data are available upon request directed to S.N.P. (spyros@chemeng.upatras.gr).

## Supplementary Materials

The following supporting information can be downloaded at: https://www.mdpi.com/article/10.3390/s24134110/s1. Figure S1. Scatter plots between the multiple calibration methods and the reference hourly averaged CO concentrations in Patras and Athens. Figure S2. CO timeseries (July, August, September 2023) in Athens using hourly averaged concentrations. Figure S3. CO timeseries (October, November, December 2023) in Athens using hourly averaged concentrations. Figure S4. CO timeseries (July, August, September 2023) in Patras using hourly averaged concentrations. Figure S5. CO timeseries (October, November, December 2023) in Patras using hourly averaged concentrations. Figure S6. Scatter plots between the multiple calibration methods and the reference hourly averaged O_3_ concentrations at the second and third sites. Figure S7. O_3_ timeseries (July, August, September 2023) in Athens using hourly averaged concentrations. Figure S8. O_3_ timeseries (October, November, December 2023) in Athens using hourly averaged concentrations. Figure S9. O_3_ timeseries (July, August, September 2023) in Patras using hourly averaged concentrations. Figure S10. O_3_ timeseries (October, November, December 2023) in Patras using hourly averaged concentrations. Figure S11. Scatter plots between the multiple calibration methods and the reference hourly averaged NO_2_ concentrations at the second and third sites. Figure S12. NO_2_ timeseries (July, August, September 2023) in Athens using hourly averaged concentrations. Figure S13. NO_2_ timeseries (September, October, November 2023) in Athens using hourly averaged concentrations. Figure S14. NO_2_ timeseries (July, August, September, October 2023) in Patras using hourly averaged concentrations. Figure S15. Scatter plots between the multiple calibration methods and the reference hourly averaged NO concentrations at the second and third sites. Figure S16. NO timeseries (July, August, September, October 2023) in Patras using hourly averaged concentrations. Table S1. CO performance metrics across multiple seasons (2023). Table S2. O_3_ performance metrics across multiple seasons (2023). Table S3. NO_2_ performance metrics across multiple seasons (2023). Table S4. NO performance metrics across multiple seasons (2023). Table S5. Comparison against the litarature. LR: Linear Regression; HDMR: High-Dimensional Model Representation; LSTM: Long-Short Term Memory network; MEq: Manufacturer’s Equations; SVR: Support Vector Regression; KNN: K-nearest neighbors; CNN: Convolutional Neural Network. References [5,11,13,14,17,25,27,31,36] are cited in the supplementary materials.

## References

[1] Almetwally A.A., Bin-Jumah M., Allam A.A. (2020). Ambient Air Pollution and Its Influence on Human Health and Welfare: An Overview. Environ. Sci. Pollut. Res..

[2] Zhang J.J., Wei Y., Fang Z. (2019). Ozone Pollution: A Major Health Hazard Worldwide. Front. Immunol..

[3] Alahmad B., Khraishah H., Althalji K., Borchert W., Al-Mulla F., Koutrakis P. (2023). Connections Between Air Pollution, Climate Change, and Cardiovascular Health. Can. J. Cardiol..

[4] Kim J., Shusterman A.A., Lieschke K.J., Newman C., Cohen R.C. (2018). The BErkeley Atmospheric CO_2_ Observation Network: Field Calibration and Evaluation of Low-Cost Air Quality Sensors. Atmos. Meas. Tech..

[5] Zimmerman N., Presto A.A., Kumar S.P.N., Gu J., Hauryliuk A., Robinson E.S., Robinson A.L., Subramanian R. (2018). A Machine Learning Calibration Model Using Random Forests to Improve Sensor Performance for Lower-Cost Air Quality Monitoring. Atmos. Meas. Tech..

[6] Cui H., Zhang L., Li W., Yuan Z., Wu M., Wang C., Ma J., Li Y. (2021). A New Calibration System for Low-Cost Sensor Network in Air Pollution Monitoring. Atmos. Pollut. Res..

[7] Masson N., Piedrahita R., Hannigan M. (2015). Approach for Quantification of Metal Oxide Type Semiconductor Gas Sensors Used for Ambient Air Quality Monitoring. Sens. Actuators B Chem..

[8] Esposito E., Salvato M., Vito S.D., Fattoruso G., Castell N., Karatzas K., Francia G.D., Leone A., Forleo A., Francioso L., Capone S., Siciliano P., Di Natale C. (2018). Assessing the Relocation Robustness of on Field Calibrations for Air Quality Monitoring Devices. Sensors and Microsystems.

[9] Borrego C., Ginja J., Coutinho M., Ribeiro C., Karatzas K., Sioumis T., Katsifarakis N., Konstantinidis K., De Vito S., Esposito E. (2018). Assessment of Air Quality Microsensors versus Reference Methods: The EuNetAir Joint Exercise—Part II. Atmos. Environ..

[10] Zauli-Sajani S., Marchesi S., Pironi C., Barbieri C., Poluzzi V., Colacci A. (2021). Assessment of Air Quality Sensor System Performance after Relocation. Atmos. Pollut. Res..

[11] Han P., Mei H., Liu D., Zeng N., Tang X., Wang Y., Pan Y. (2021). Calibrations of Low-Cost Air Pollution Monitoring Sensors for CO, NO_2_, O_3_, and SO_2_. Sensors.

[12] Zuidema C., Schumacher C.S., Austin E., Carvlin G., Larson T.V., Spalt E.W., Zusman M., Gassett A.J., Seto E., Kaufman J.D. (2021). Deployment, Calibration, and Cross-Validation of Low-Cost Electrochemical Sensors for Carbon Monoxide, Nitrogen Oxides, and Ozone for an Epidemiological Study. Sensors.

[13] Bigi A., Mueller M., Grange S.K., Ghermandi G., Hueglin C. (2018). Performance of NO, NO_2_ Low Cost Sensors and Three Calibration Approaches within a Real World Application. Atmos. Meas. Tech..

[14] Vajs I., Drajic D., Gligoric N., Radovanovic I., Popovic I. (2021). Developing Relative Humidity and Temperature Corrections for Low-Cost Sensors Using Machine Learning. Sensors.

[15] Mead M.I., Popoola O.A.M., Stewart G.B., Landshoff P., Calleja M., Hayes M., Baldovi J.J., McLeod M.W., Hodgson T.F., Dicks J. (2013). The Use of Electrochemical Sensors for Monitoring Urban Air Quality in Low-Cost, High-Density Networks. Atmos. Environ..

[16] Spinelle L., Gerboles M., Villani M.G., Aleixandre M., Bonavitacola F. (2015). Field Calibration of a Cluster of Low-Cost Available Sensors for Air Quality Monitoring. Part A: Ozone and Nitrogen Dioxide. Sens. Actuators B Chem..

[17] Cross E.S., Williams L.R., Lewis D.K., Magoon G.R., Onasch T.B., Kaminsky M.L., Worsnop D.R., Jayne J.T. (2017). Use of Electrochemical Sensors for Measurement of Air Pollution: Correcting Interference Response and Validating Measurements. Atmos. Meas. Tech..

[18] Mueller M., Meyer J., Hueglin C. (2017). Design of an Ozone and Nitrogen Dioxide Sensor Unit and Its Long-Term Operation within a Sensor Network in the City of Zurich. Atmos. Meas. Tech..

[19] Mijling B., Jiang Q., De Jonge D., Bocconi S. (2018). Field Calibration of Electrochemical NO_2_ Sensors in a Citizen Science Context. Atmos. Meas. Tech..

[20] Bart M., Williams D.E., Ainslie B., McKendry I., Salmond J., Grange S.K., Alavi-Shoshtari M., Steyn D., Henshaw G.S. (2014). High Density Ozone Monitoring Using Gas Sensitive Semi-Conductor Sensors in the Lower Fraser Valley, British Columbia. Environ. Sci. Technol..

[21] Masson N., Piedrahita R., Hannigan M. (2015). Quantification Method for Electrolytic Sensors in Long-Term Monitoring of Ambient Air Quality. Sensors.

[22] Topalović D.B., Davidović M.D., Jovanović M., Bartonova A., Ristovski Z., Jovašević-Stojanović M. (2019). In Search of an Optimal In-Field Calibration Method of Low-Cost Gas Sensors for Ambient Air Pollutants: Comparison of Linear, Multilinear and Artificial Neural Network Approaches. Atmos. Environ..

[23] Pang X., Shaw M.D., Lewis A.C., Carpenter L.J., Batchellier T. (2017). Electrochemical Ozone Sensors: A Miniaturised Alternative for Ozone Measurements in Laboratory Experiments and Air-Quality Monitoring. Sens. Actuators B Chem..

[24] Moltchanov S., Levy I., Etzion Y., Lerner U., Broday D.M., Fishbain B. (2015). On the Feasibility of Measuring Urban Air Pollution by Wireless Distributed Sensor Networks. Sci. Total Environ..

[25] Papaconstantinou R., Demosthenous M., Bezantakos S., Hadjigeorgiou N., Costi M., Stylianou M., Symeou E., Savvides C., Biskos G. (2023). Field Evaluation of Low-Cost Electrochemical Air Quality Gas Sensors under Extreme Temperature and Relative Humidity Conditions. Atmos. Meas. Tech..

[26] Jiao W., Hagler G., Williams R., Sharpe R., Brown R., Garver D., Judge R., Caudill M., Rickard J., Davis M. (2016). Community Air Sensor Network (CAIRSENSE) Project: Evaluation of Low-Costsensor Performance in a Suburban Environment in the Southeastern UnitedStates. Atmos. Meas. Tech..

[27] Apostolopoulos I.D., Fouskas G., Pandis S.N. (2023). Field Calibration of a Low-Cost Air Quality Monitoring Device in an Urban Background Site Using Machine Learning Models. Atmosphere.

[28] Kim H., Müller M., Henne S., Hüglin C. (2022). Long-Term Behavior and Stability of Calibration Models for NO and NO_2_ Low-Cost Sensors. Atmos. Meas. Tech..

[29] Apostolopoulos I.D., Fouskas G., Pandis S.N., Perakovic D., Knapcikova L. (2022). An IoT Integrated Air Quality Monitoring Device Based on Microcomputer Technology and Leading Industry Low-Cost Sensor Solutions. Future Access Enablers for Ubiquitous and Intelligent Infrastructures.

[30] Piedrahita R., Xiang Y., Masson N., Ortega J., Collier A., Jiang Y., Li K., Dick R.P., Lv Q., Hannigan M. (2014). The next Generation of Low-Cost Personal Air Quality Sensors for Quantitative Exposure Monitoring. Atmos. Meas. Tech..

[31] Ariyaratne R., Elangasinghe M.A., Zamora M.L., Karunaratne D.G.G.P., Manipura A., Jinadasa K.B.S.N., Abayalath K.H.N. (2023). Understanding the Effect of Temperature and Relative Humidity on Sensor Sensitivities in Field Environments and Improving the Calibration Models of Multiple Electrochemical Carbon Monoxide (CO) Sensors in a Tropical Environment. Sens. Actuators B Chem..

[32] Tryner J., Phillips M., Quinn C., Neymark G., Wilson A., Jathar S.H., Carter E., Volckens J. (2021). Design and Testing of a Low-Cost Sensor and Sampling Platform for Indoor Air Quality. Build. Environ..

[33] Casey J.G., Collier-Oxandale A., Hannigan M. (2019). Performance of Artificial Neural Networks and Linear Models to Quantify 4 Trace Gas Species in an Oil and Gas Production Region with Low-Cost Sensors. Sens. Actuators B Chem..

[34] Kalkavouras P., Grivas G., Stavroulas I., Petrinoli K., Bougiatioti A., Liakakou E., Gerasopoulos E., Mihalopoulos N. (2024). Source Apportionment of Fine and Ultrafine Particle Number Concentrations in a Major City of the Eastern Mediterranean. Sci. Total Environ..

[35] Grivas G., Athanasopoulou E., Kakouri A., Bailey J., Liakakou E., Stavroulas I., Kalkavouras P., Bougiatioti A., Kaskaoutis D., Ramonet M. (2020). Integrating in Situ Measurements and City Scale Modelling to Assess the COVID–19 Lockdown Effects on Emissions and Air Quality in Athens, Greece. Atmosphere.

[36] Zuidema C., Afshar-Mohajer N., Tatum M., Thomas G., Peters T., Koehler K. (2019). Efficacy of paired electrochemical sensors for measuring ozone concentrations. J. Occup. Environ. Hyg..

