# Supplementary material for: Calibration and Inter-Unit Consistency Assessment of an Electrochemical Sensor System Using Machine Learning
Source: Sensors (Basel). 2024 Jun 25;24(13):4110. doi: 10.3390/s24134110 (PMC11244084; doi:10.3390/s24134110)
Supplement: Supplementary file 1 [file sensors-24-04110-s001.zip › sensors-3040812-supplementary.pdf]

Supplementary Material: Calibration and Inter-Unit Consistency Assessment of an Electrochemical Sensor System Using Machine Learning

Ioannis D. Apostolopoulos [1], Silas Androulakis [1,2], Panayiotis Kalkavouras [3,4], George Fouskas [1] and Spyros N. Pandis [1,2,*]

[1] Institute of Chemical Engineering Sciences (ICE-HT), Foundation for Research and Technology Hellas (FORTH), 26504 Patras, Greece; japostol@iceht.forth.gr (I.D.A.); silas.androul@gmail.com (S.A.); fouskas@iceht.forth.gr (G.F.)
[2] Department of Chemical Engineering, University of Patras, 26504 Patras, Greece
[3] Institute for Environmental Research & Sustainable Development, National Observatory of Athens, 11810 Athens, Greece; pkalkavouras@noa.gr
[4] Department of Environment, University of the Aegean, 81400 Mytilene, Greece
* Correspondence: spyros@chemeng.upatras.gr

1. Carbon monoxide

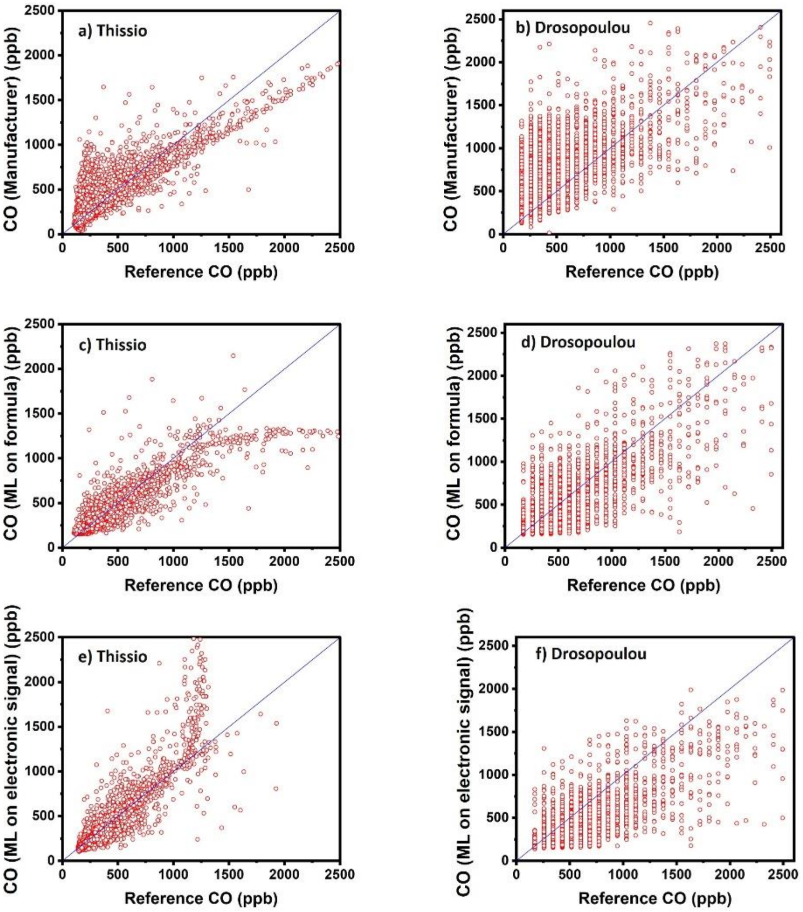

Figure S1. Scatter plots between the multiple calibration methods and the reference hourly averaged CO concentrations in Patras and Athens.

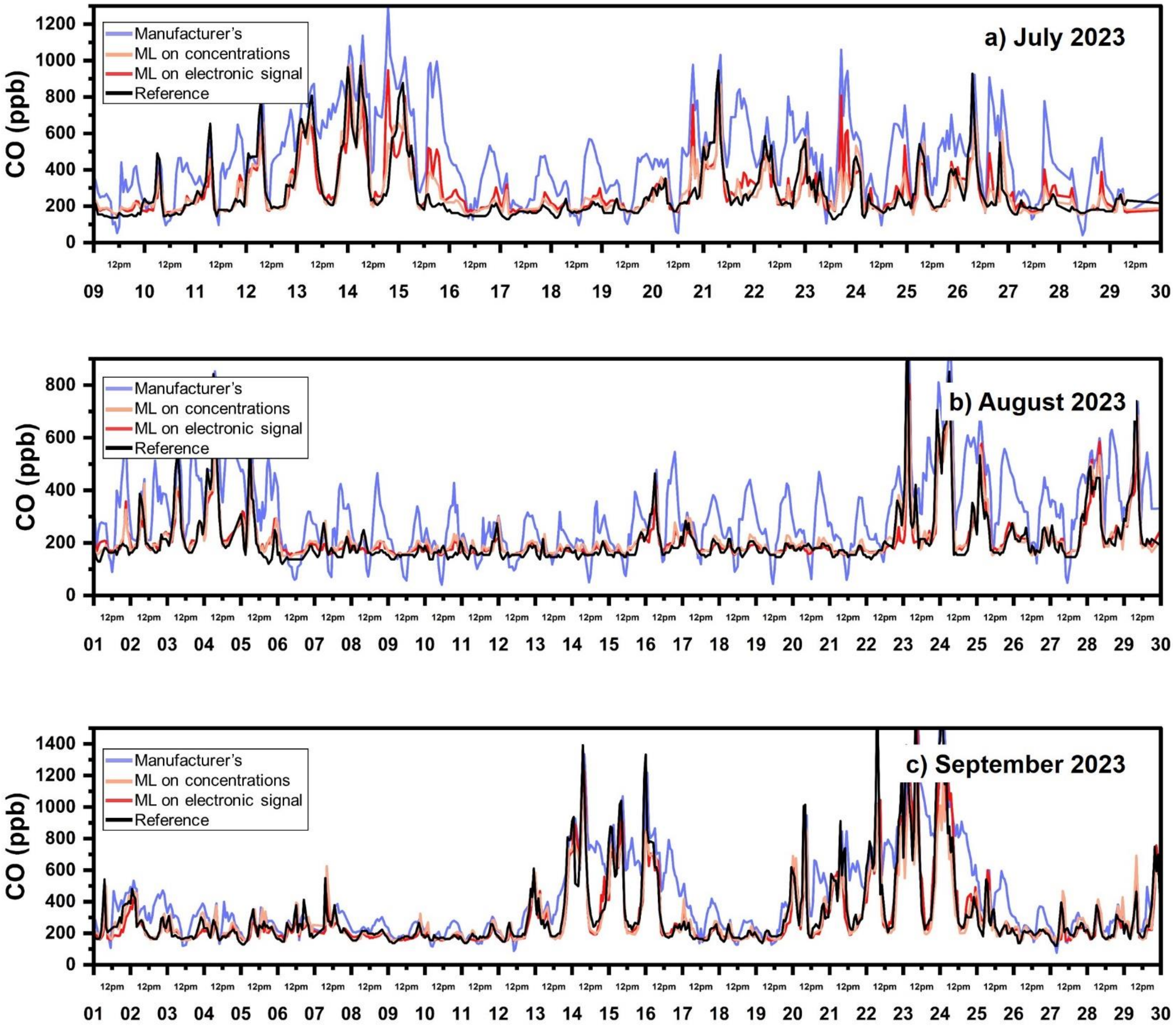

Figure S2. CO timeseries (July, August, September 2023) in Athens using hourly averaged concentrations.

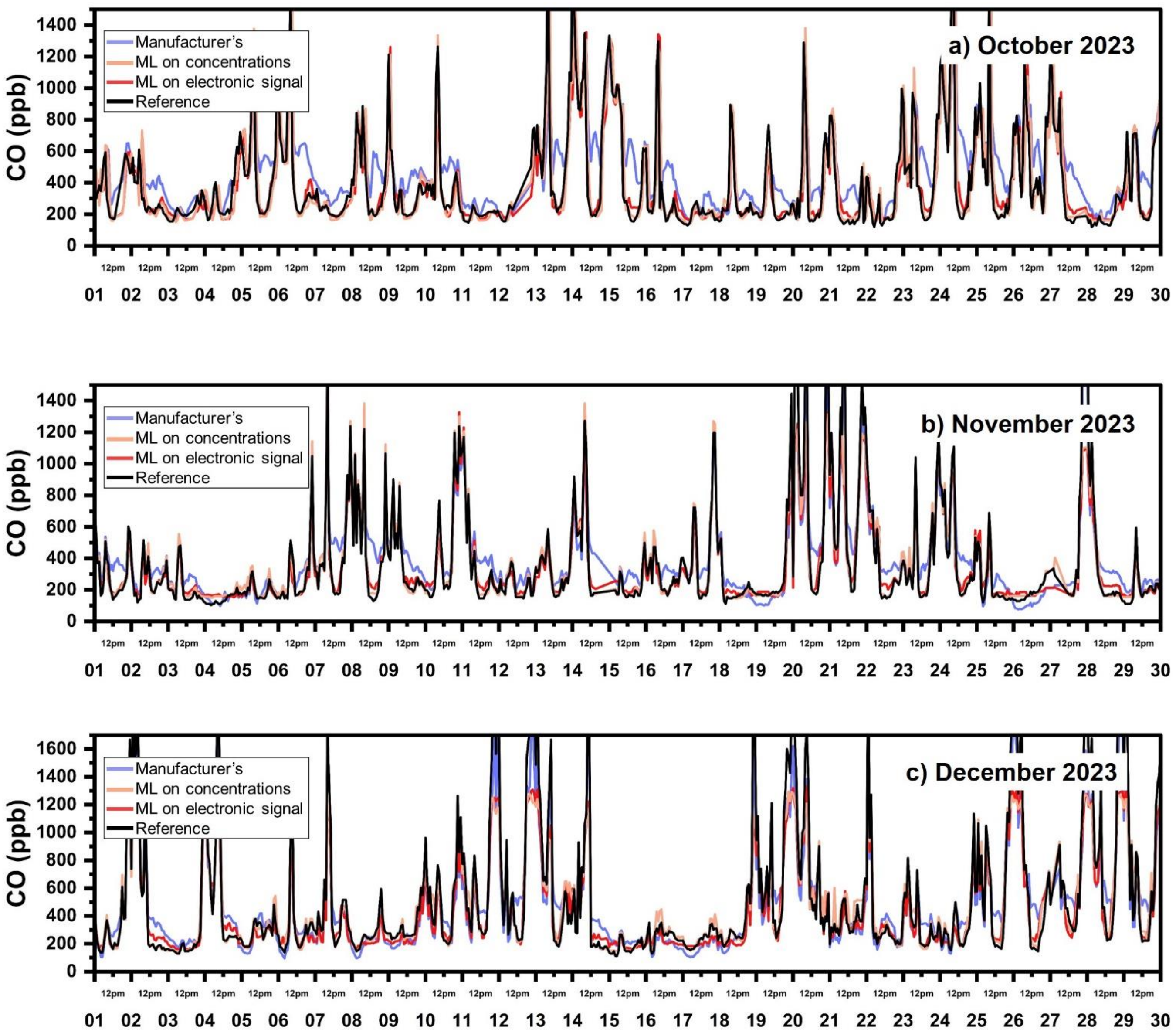

Figure S3. CO timeseries (October, November, December 2023) in Athens using hourly averaged concentrations.

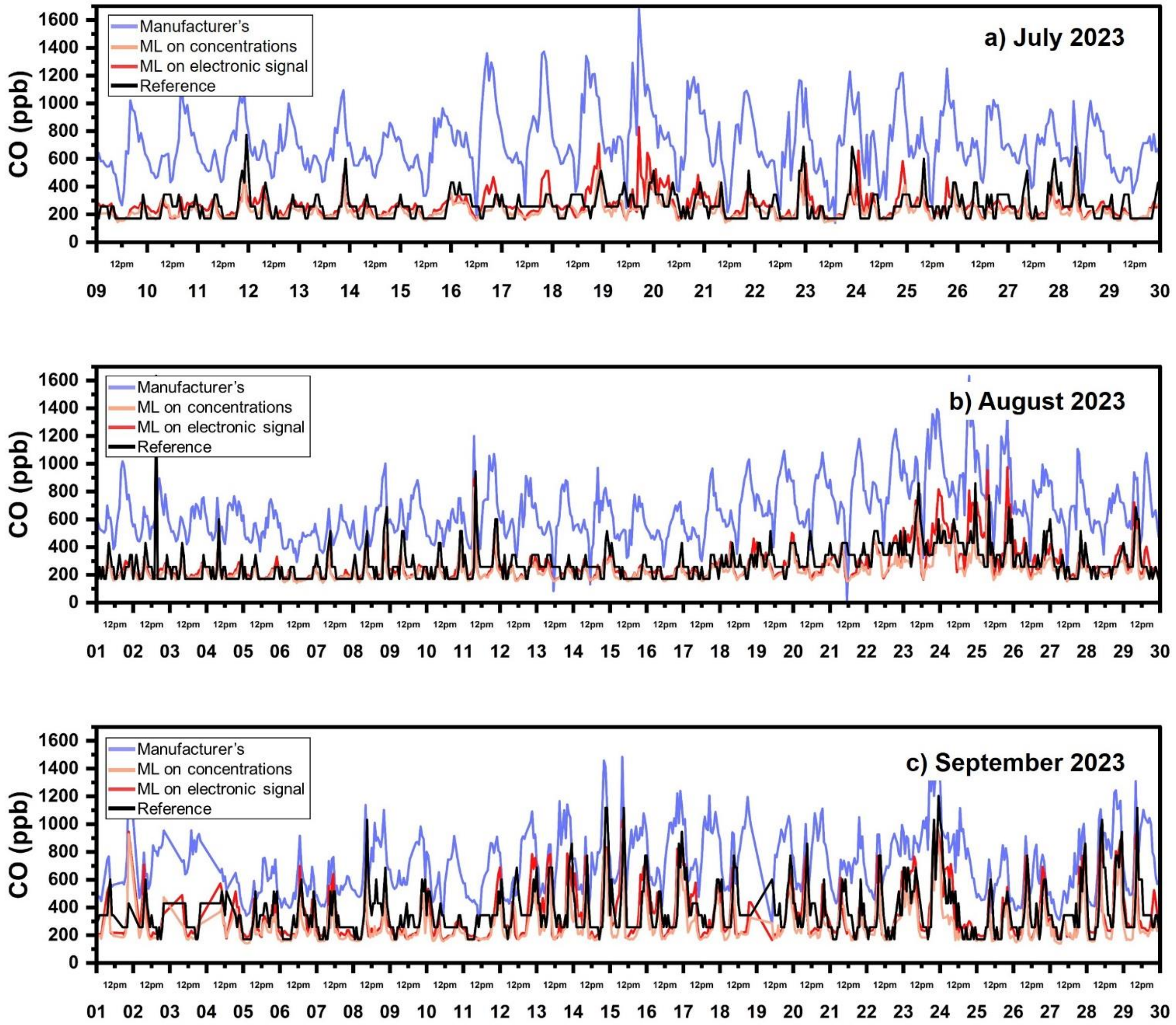

Figure S4. CO timeseries (July, August, September 2023) in Patras using hourly averaged concentrations.

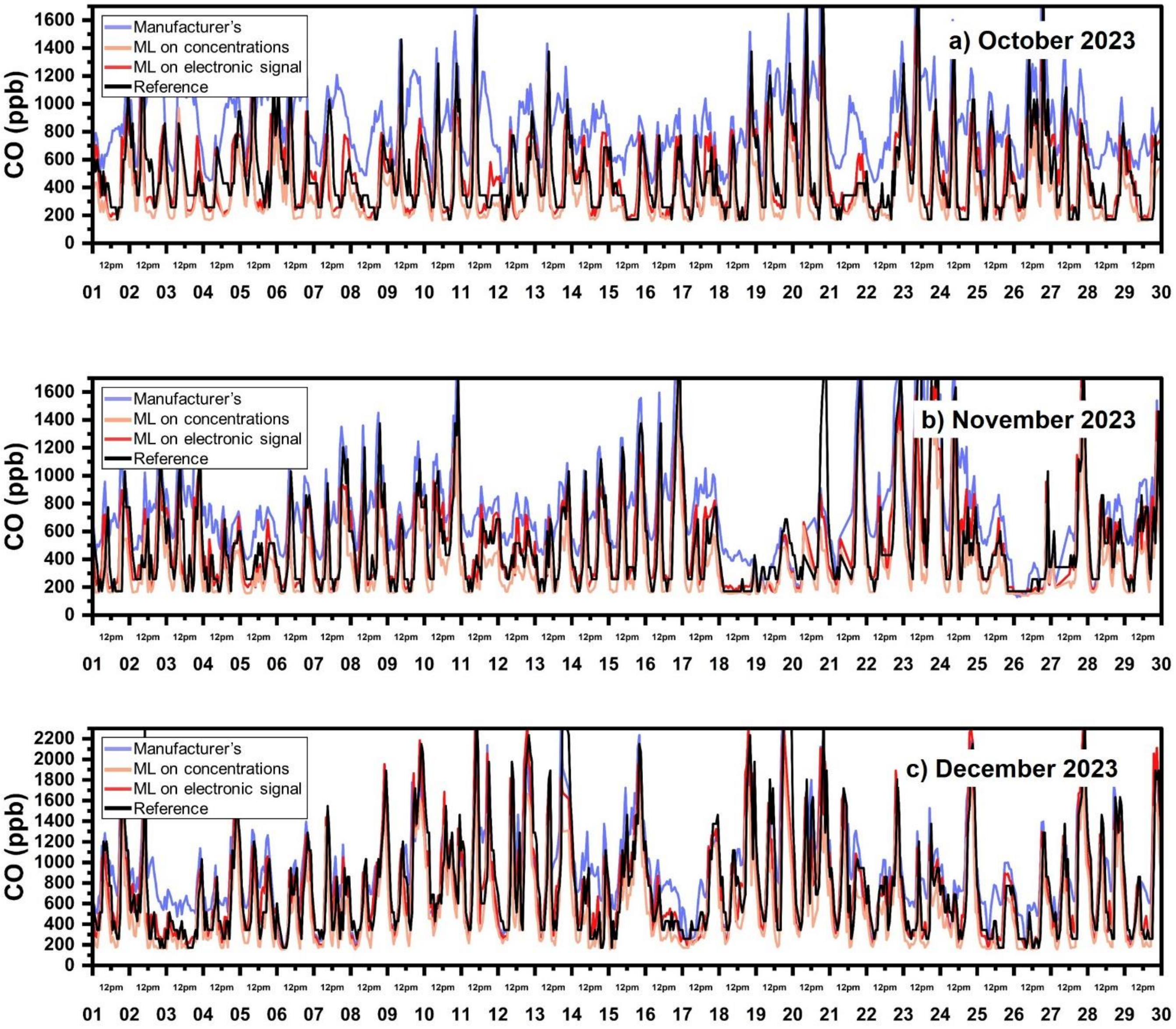

Figure S5. CO timeseries (October, November, December 2023) in Patras using hourly averaged concentrations.

2. Ozone

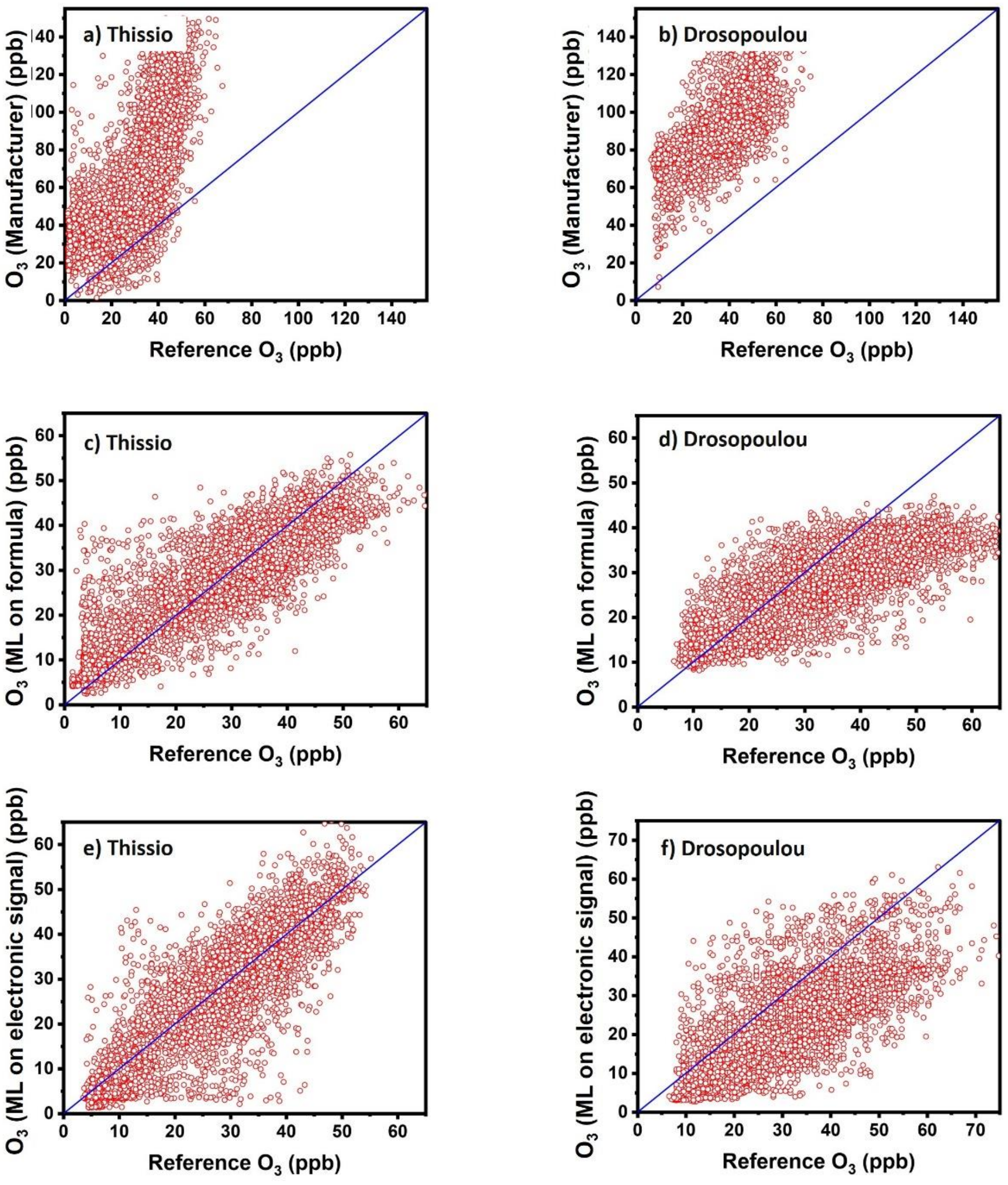

Figure S6. Scatter plots between the multiple calibration methods and the reference hourly averaged O_3 concentrations at the second and third sites.

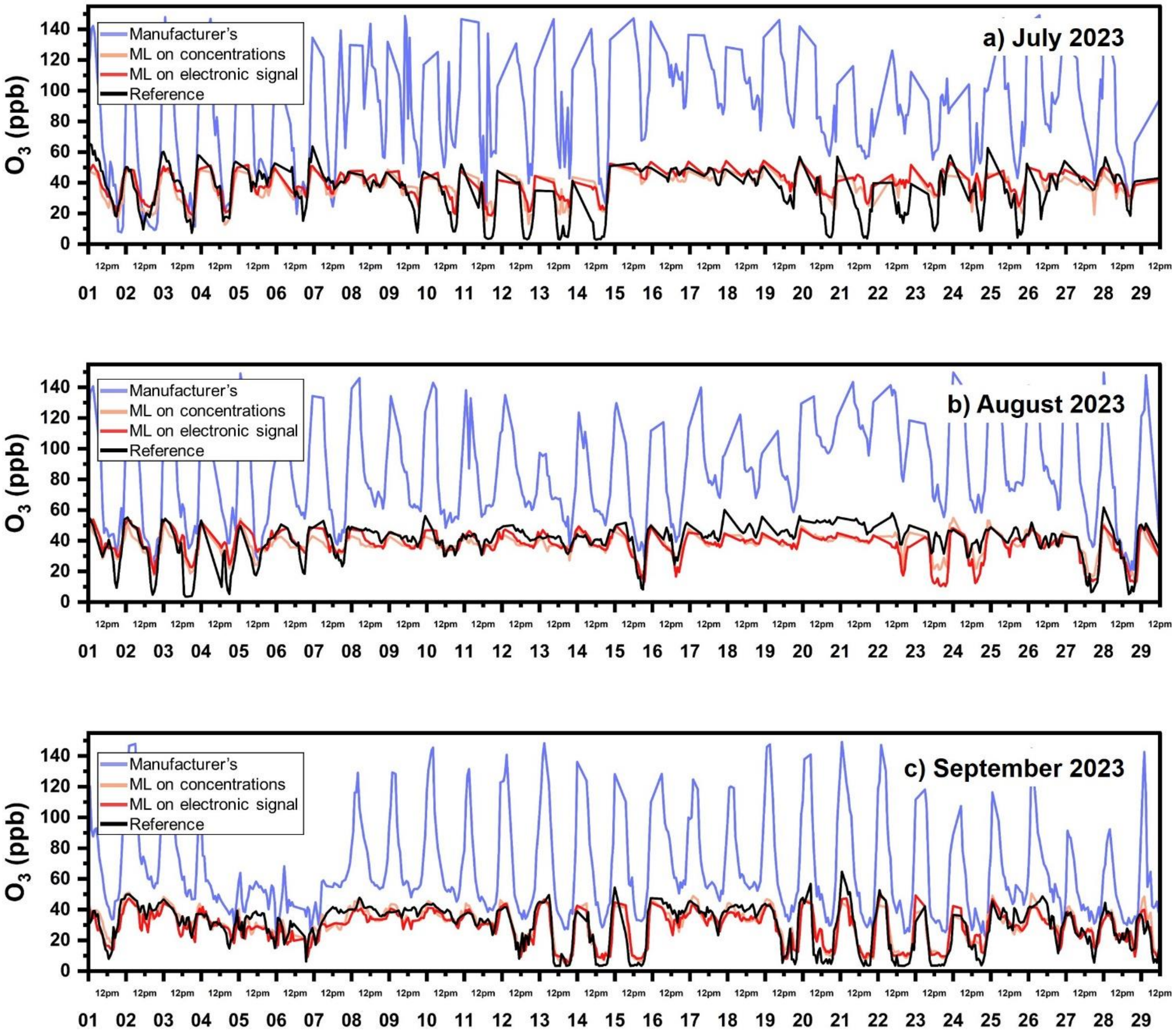

Figure S7. O_3 timeseries (July, August, September 2023) in Athens using hourly averaged concentrations.

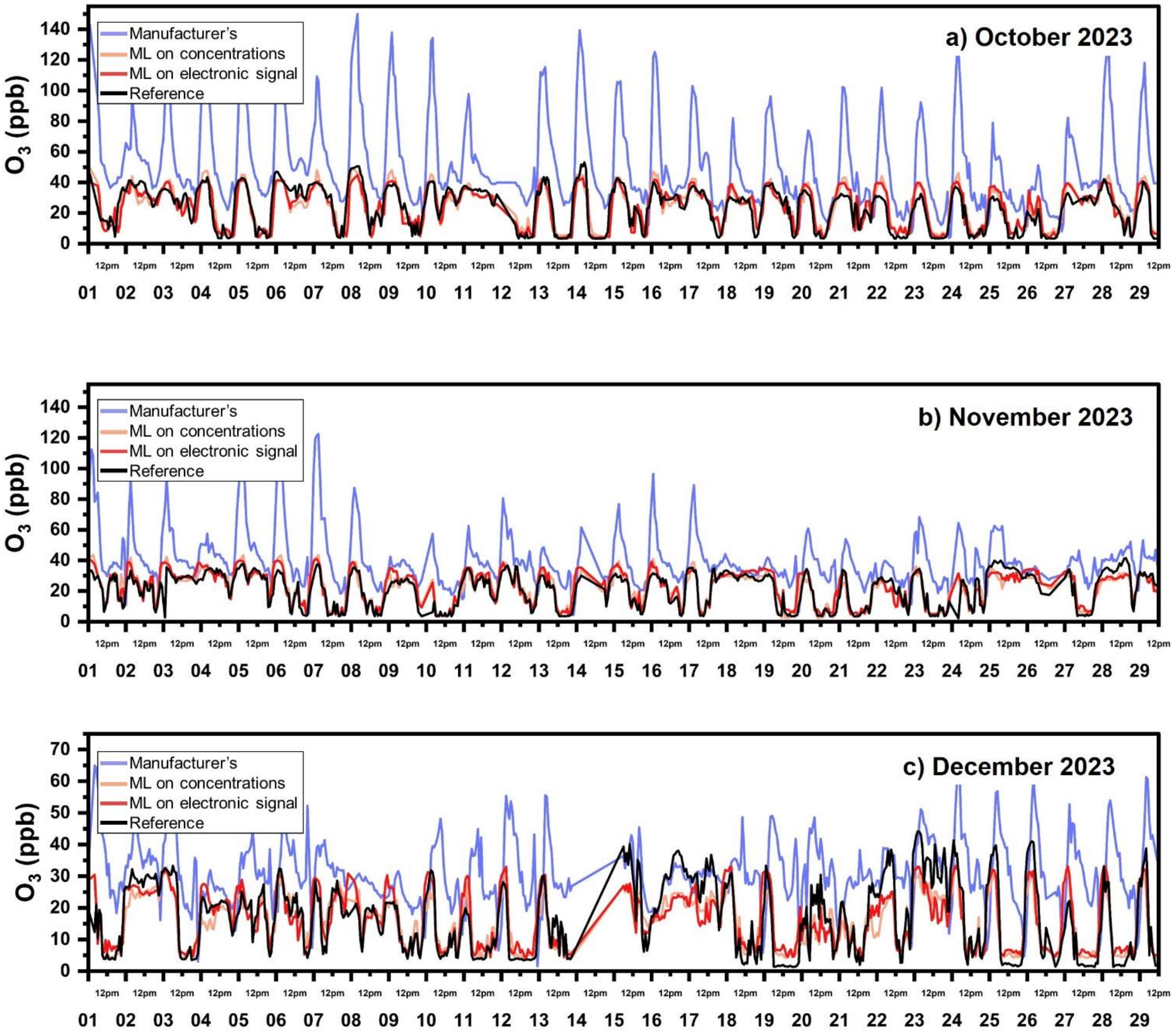

Figure S8. O_3 timeseries (October, November, December 2023) in Athens using hourly averaged concentrations.

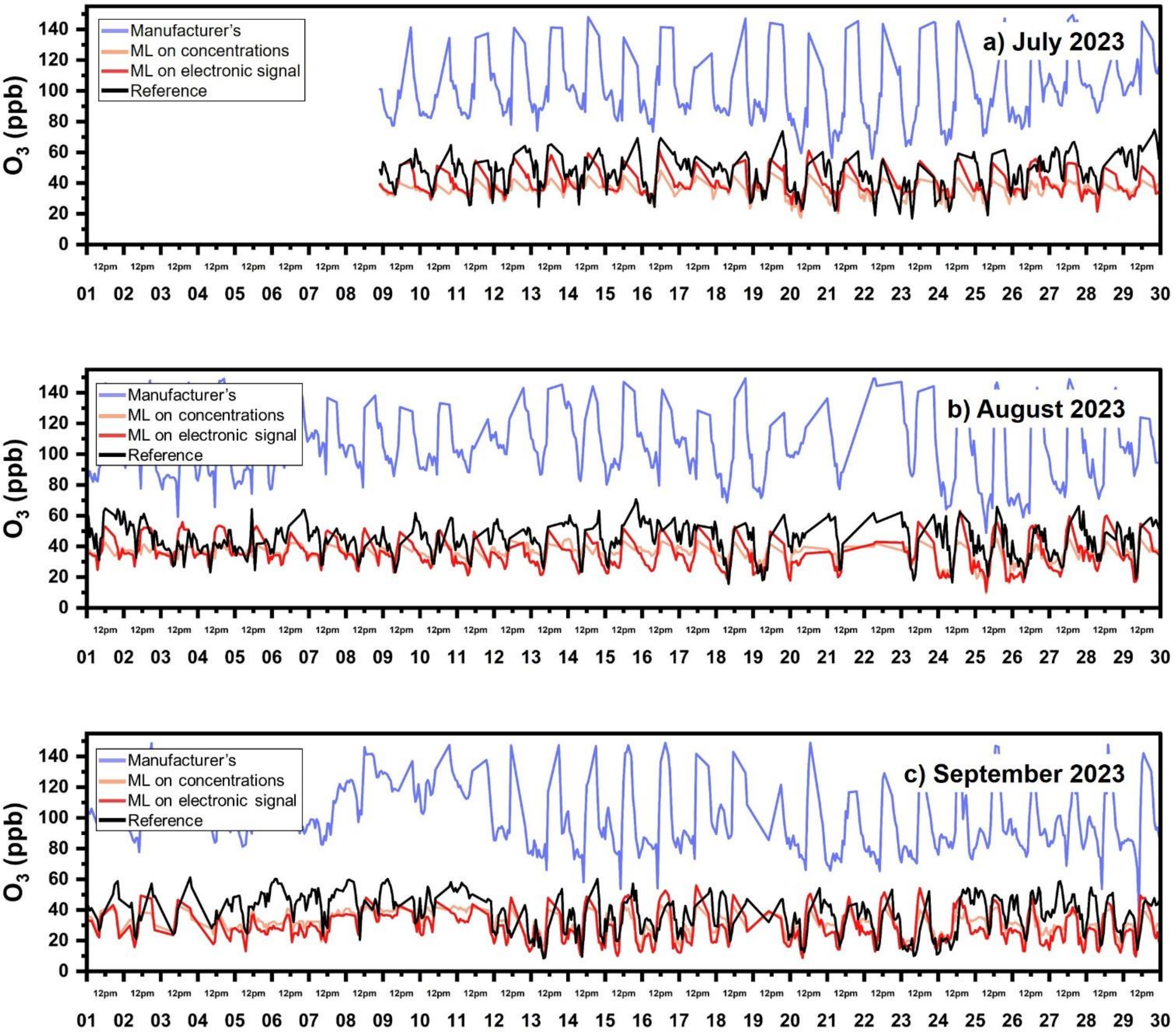

Figure S9. O_3 timeseries (July, August, September 2023) in Patras using hourly averaged concentrations.

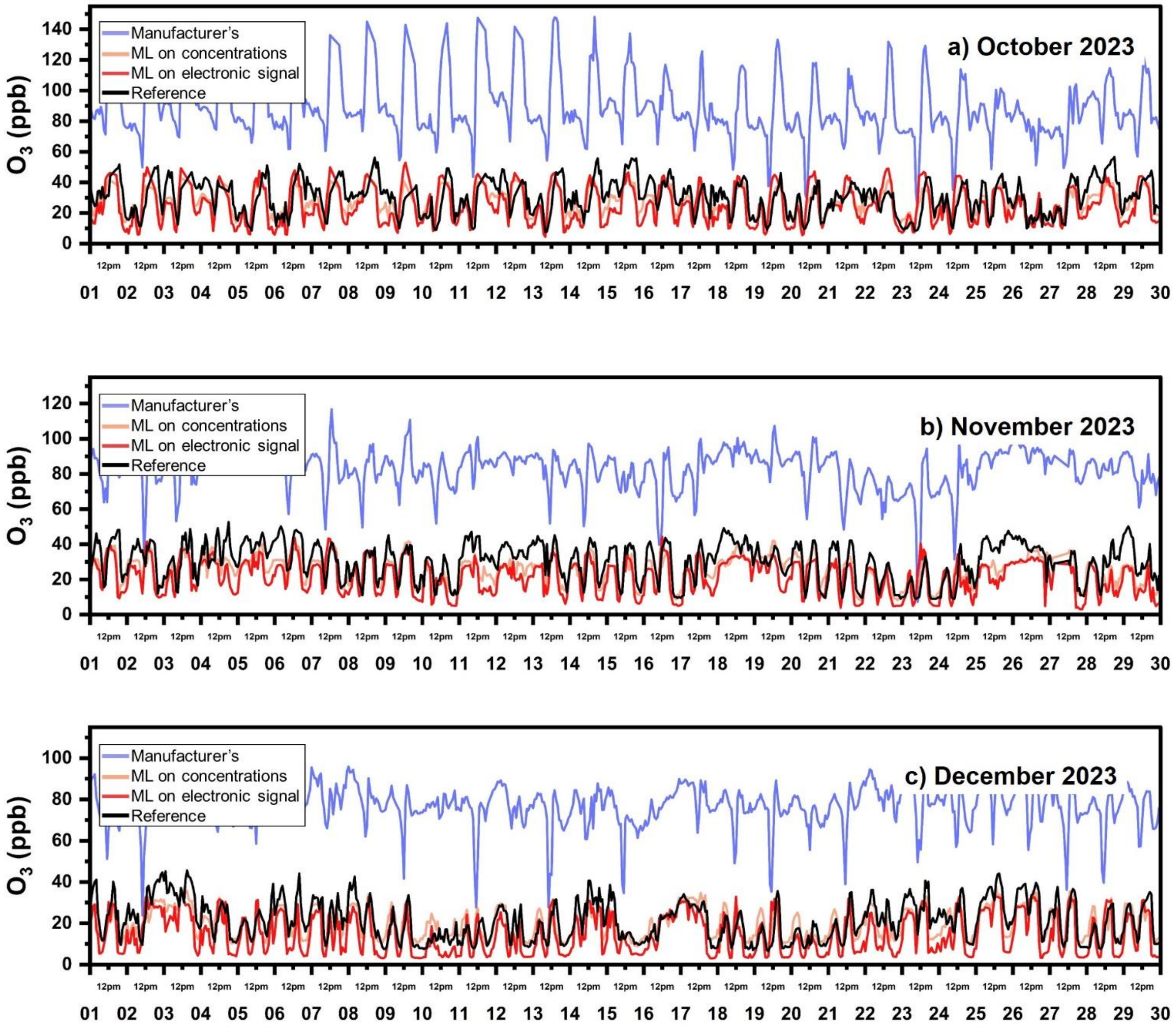

Figure S10. O_3 timeseries (October, November, December 2023) in Patras using hourly averaged concentrations.

3. Nitrogen Dioxide

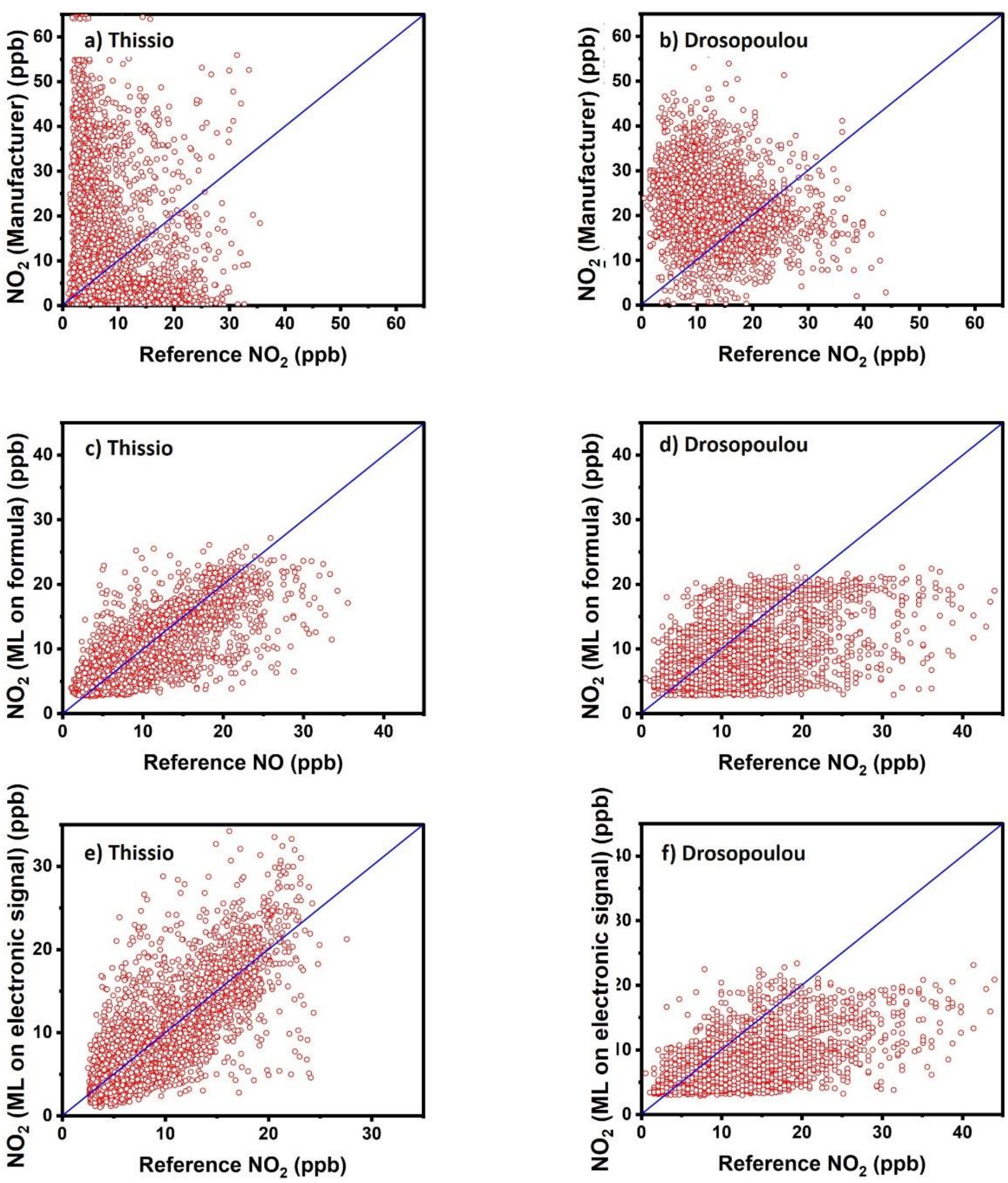

Figure S11 Scatter plots between the multiple calibration methods and the reference hourly averaged NO_2 concentrations at the second and third sites.

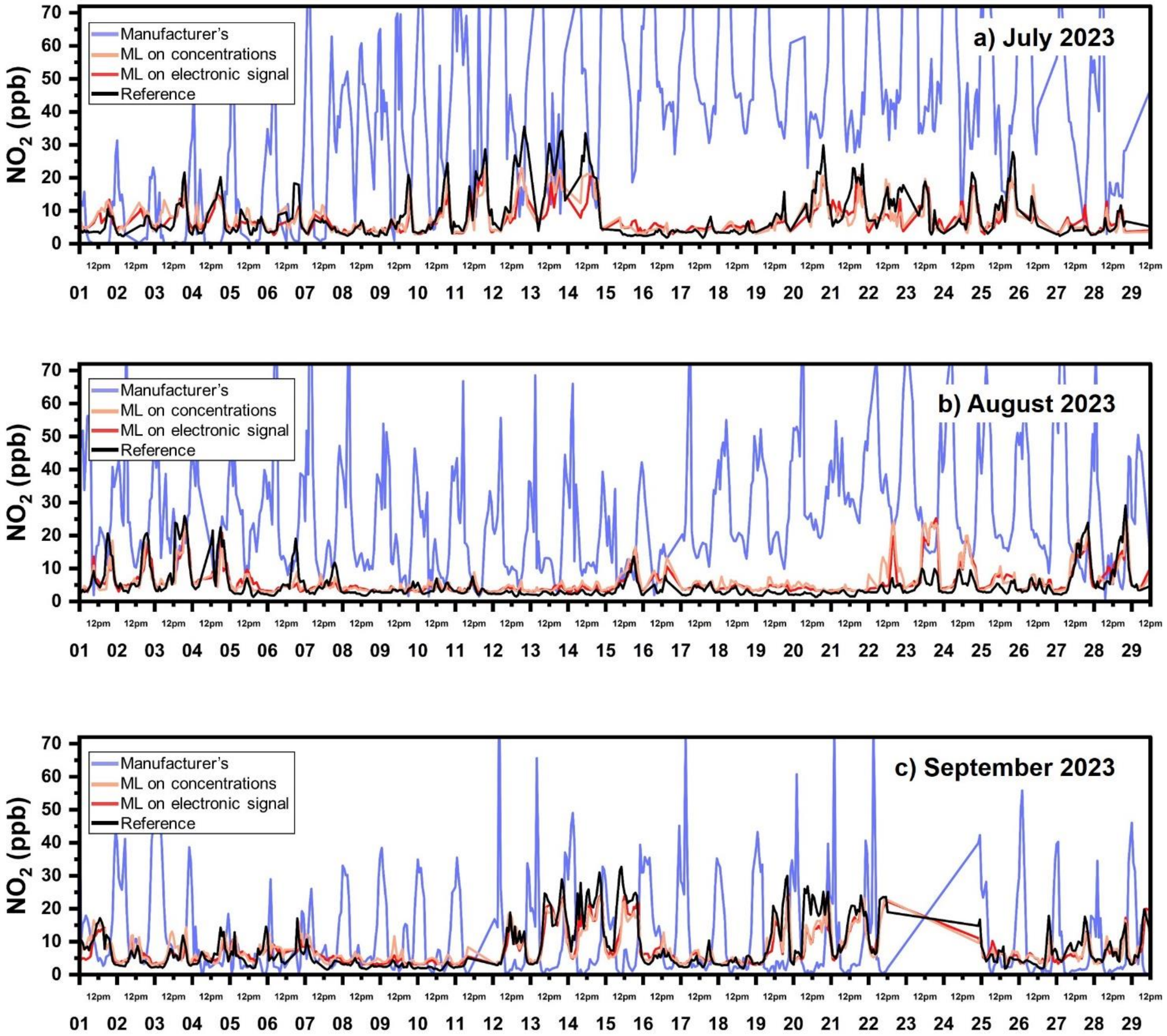

Figure S12. NO_2 timeseries (July, August, September 2023) in Athens using hourly averaged concentrations.

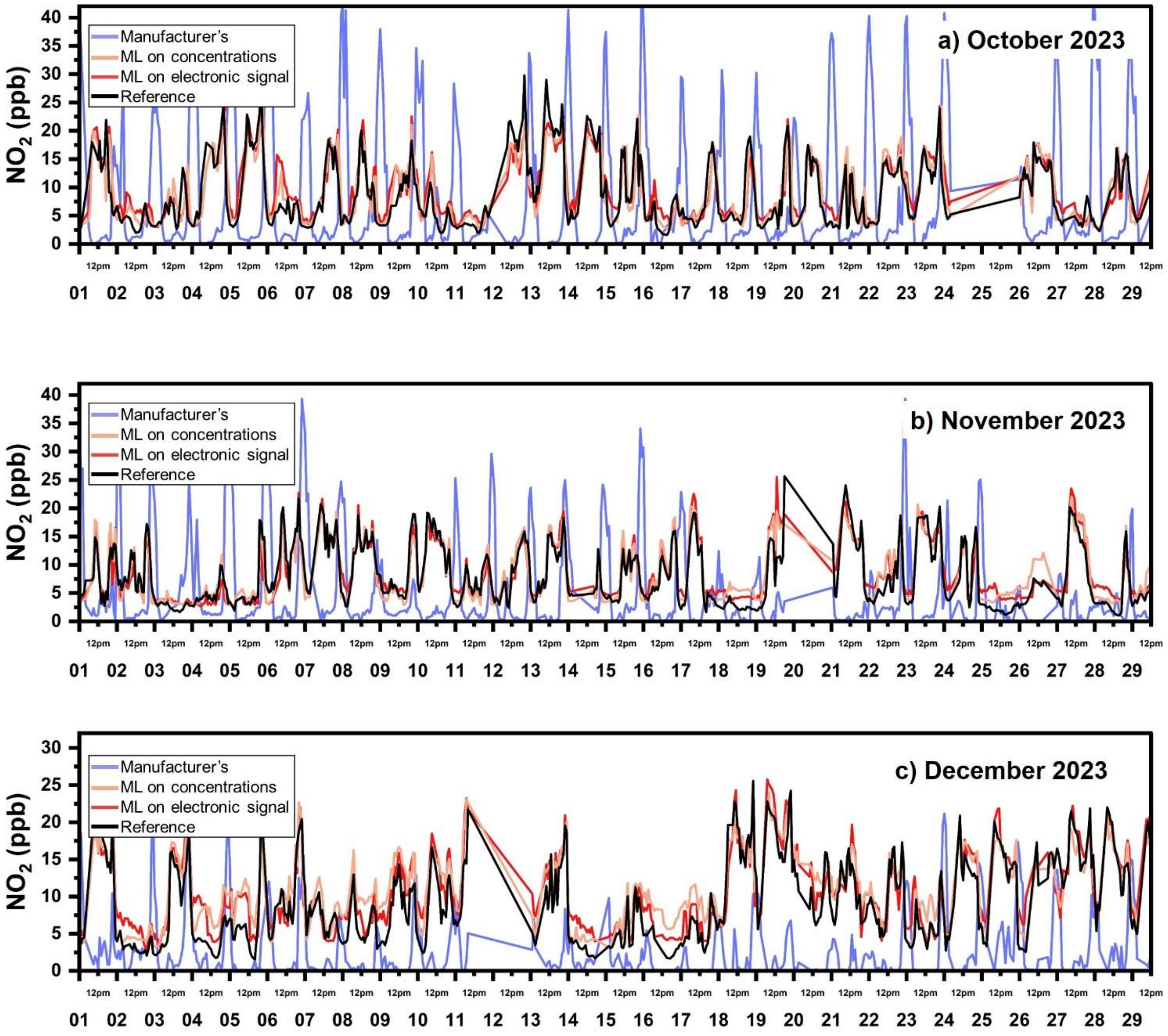

Figure S13. NO_2 timeseries (September, October, November 2023) in Athens using hourly averaged concentrations.

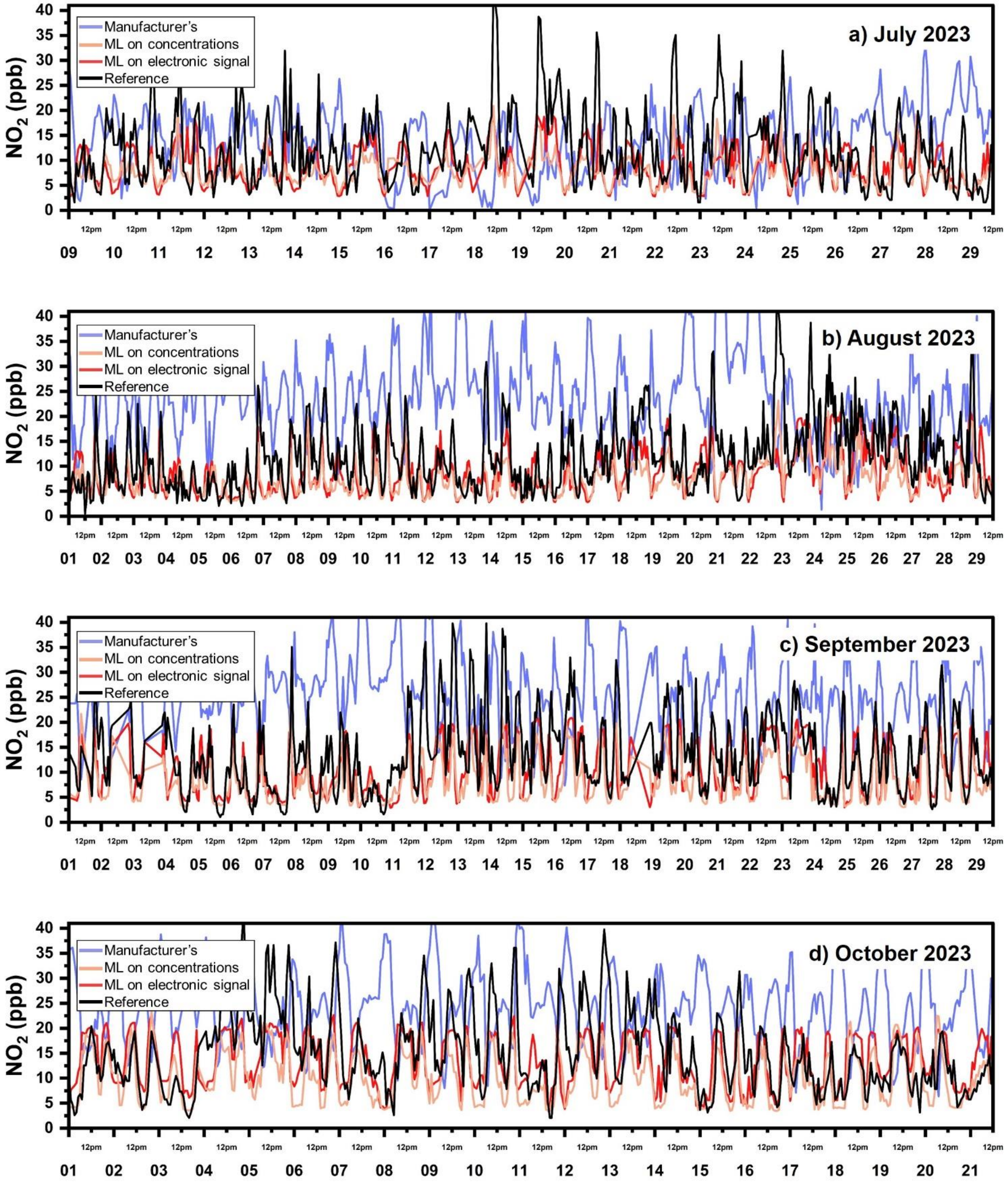

Figure S14. NO_2 timeseries (July, August, September, October 2023) in Patras using hourly averaged concentrations.

4. Nitric Oxide

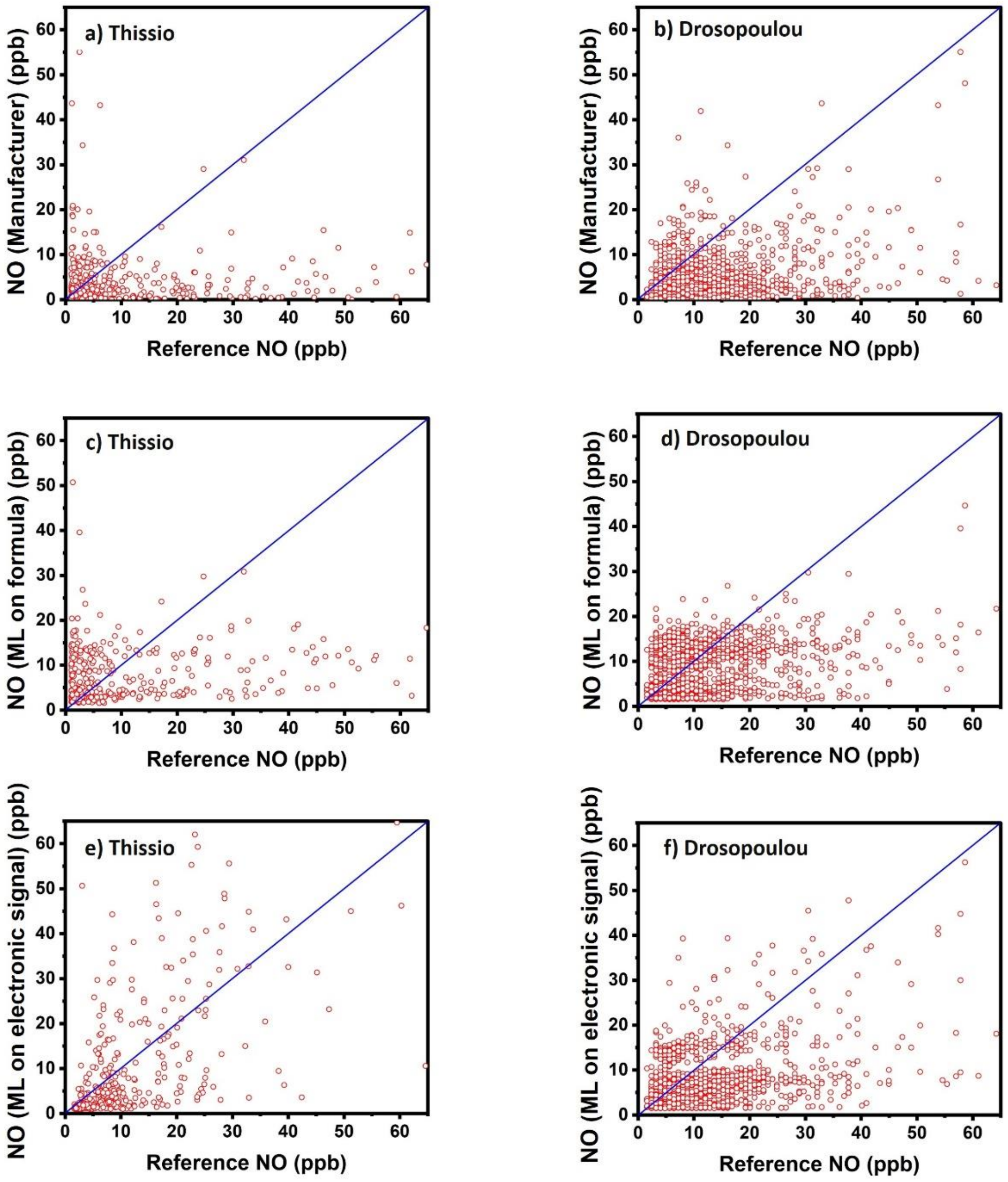

Figure S15 Scatter plots between the multiple calibration methods and the reference hourly averaged NO concentrations at the second and third sites.

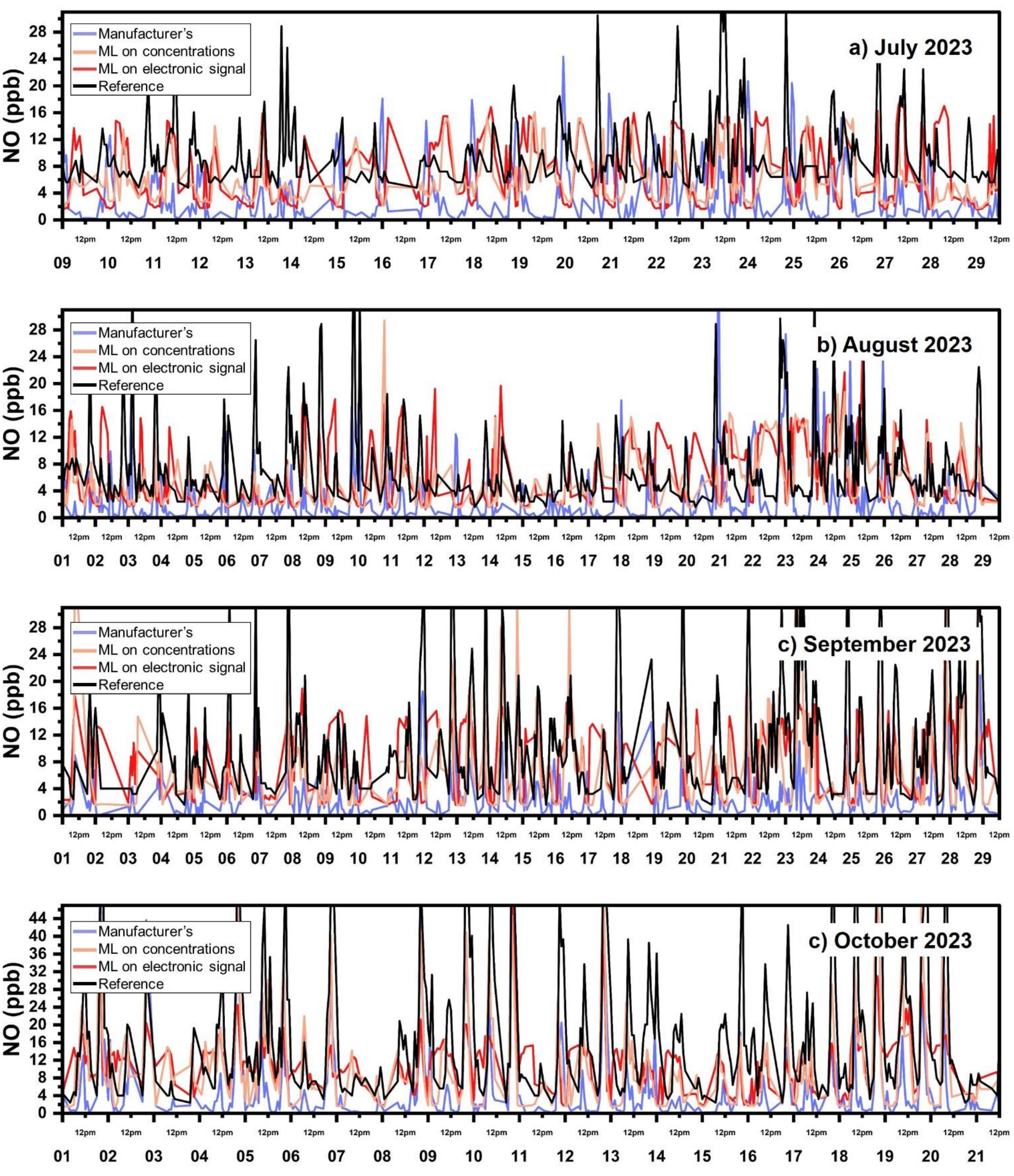

Figure S16. NO timeseries (July, August, September, October 2023) in Patras using hourly averaged concentrations.

Table S1. CO performance metrics across multiple seasons (2023)

	Summer				Autumn				Winter			
	Athens		Patras		Athens		Patras		Athens		Patras	
	nME	R^2	nME	R^2	nME	R^2	nME	R^2	nME	R^2	nME	R^2
Manufacturer's equations	0.76	0.52	1.25	0.34	0.48	0.72	1.07	0.45	0.44	0.76	1.02	0.42
ML on concentrations	0.23	0.73	0.38	0.57	0.17	0.83	0.28	0.68	0.17	0.83	0.26	0.69
ML on electronic signal	0.21	0.75	0.36	0.6	0.14	0.84	0.25	0.71	0.14	0.85	0.14	0.72

Table S2. O_3 performance metrics across multiple seasons (2023)

	Summer				Autumn				Winter			
	Athens		Patras		Athens		Patras		Athens		Patras	
	nME	R^2	nME	R^2	nME	R^2	nME	R^2	nME	R^2	nME	R^2
Manufacturer's equations	0.82	0.61	2.89	0.4	0.72	0.64	1.62	0.55	0.72	0.65	1.62	0.55
ML on concentrations	0.24	0.64	0.32	0.49	0.22	0.71	0.28	0.62	0.2	0.75	0.25	0.64
ML on electronic signal	0.22	0.7	0.39	0.52	0.2	0.72	0.28	0.62	0.19	0.81	0.26	0.69

Table S3. NO_2 performance metrics across multiple seasons (2023)

	Summer				Autumn				Winter			
	Athens		Patras		Athens		Patras		Athens		Patras	
	nME	R^2	nME	R^2	nME	R^2	nME	R^2	nME	R^2	nME	R^2
Manufacturer's equations	4.01	0.1	2.8	0.1	3.02	0.1	1.6	0.2	2.81	0.2	1.6	0.2
ML on concentrations	0.48	0.58	0.58	0.1	0.31	0.68	0.41	0.32	0.28	0.72	0.4	0.33
ML on electronic signal	0.45	0.64	0.52	0.2	0.29	0.75	0.39	0.42	0.25	0.79	0.38	0.45

Table S4. NO performance metrics across multiple seasons (2023)

	Summer				Autumn				Winter			
	Athens		Patras		Athens		Patras		Athens		Patras	
	nME	R^2	nME	R^2	nME	R^2	nME	R^2	nME	R^2	nME	R^2
Manufacturer's equations	6	0.25	1.2	0.15	4.6	0.42	0.6	0.3	4.2	0.43	0.7	0.2
ML on concentrations	1.5	0.56	0.9	0.1	0.9	0.74	0.5	0.2	0.8	0.76	0.5	0.2
ML on electronic signal	1.5	0.54	0.8	0.35	1.2	0.74	0.4	0.5	1.1	0.77	0.3	0.5

Table S5. Comparison against the litarature. LR: Linear Regression; HDMR: High-Dimensional Model Representation; LSTM: Long-Short Term Memory network; MEq: Manufacturer’s Equations; SVR: Support Vector Regression; KNN: K-nearest neighbours; CNN: Convolutional Neural Network

Study	Sensor	Period	Calibration method	Averaging	Results
**CO**					
Ariyaratne et al. [1]	CO-B4	Two-week	LR	1h	ME: 44.6 to 127.3 ppb<br>R^2: 0.94 to 0.95<br>RMSE: 52.2 to 138 ppb
Zuidema et al. [2]	CO-B4	Two-year	LR	1h	R^2: 0.97<br>RMSE: 18 ppb
Cross et al. [3]	CO-B4	4-month	HDMR	5min	ME: 24.8 ppb<br>R^2: 0.88
Zimmerman et al. [4]	CO-B4	7-month	RF	15min	R: > 0.8<br>ME: 38 ppb<br>nME: 0.14
Han et al. [5]	CO-B4	12-month	LSTM<br>RF	1h	Random Forest<br>R^2: 0.89<br>RMSE: 208 ppb
Vajs et al. [6]	CO-B4	9-month	RF<br>LR<br>ANN	1h	Random Forest<br>R_2: 0.95<br>RMSE: 60 ppb
Papaconstantinou et al. [7]	CO-B4	12-month	MEq	1h	ME: 289 ppb<br>R_2: 0.54
This study (Athens)	CO-B4	9-month	RF	1h	ME: 61 ppb<br>R^2: 0.82<br>nME: 0.16
This study (Patras)	CO-B4	9-month	RF	1h	ME: 162 ppb<br>R^2: 0.63<br>nME: 0.31
**NO**					

Zuidema et al. [2]	NO-B4	Two-year	LR	1h	R^2: 0.97<br>RMSE: 2 ppb
Cross et al. [3]	NO-B4	4-month	HDMR	5min	ME: 2.83 ppb<br>R^2: 0.84
Bigi et al. [8]	NO-B4	4-month	LR<br>SVR<br>RF	1h	Zurich<br>ME: 2.1 ppb<br>R^2: 0.95<br>RMSE: 3.3 ppb<br><br>Lausanne<br>ME: 4 ppb<br>R^2: 0.85<br>RMSE: 7.1 ppb
This study (Athens)	NO-B4	9-month	RF	1h	ME: 6 ppb<br>R^2: 0.69<br>nME: 1
This study (Patras)	NO-B4	9-month	RF	1h	ME: 5 ppb<br>R^2: 0.35<br>nME: 0.6
**NO_2**					
Cross et al. [3]	NO2-B43F	4-month	HDMR	5min	ME: 3.45 ppb<br>R^2: 0.69
Zimmerman et al. [4]	NO2-B43F	7-month	RF	15min	R: > 0.8<br>ME: 3.5 ppb<br>nME: 0.29
Han et al. [5]	NO2-B43F	12-month	LSTM<br>RF	1h	Random Forest<br>R^2: 0.85<br>RMSE: 13 ppb

Vajs et al. [6]	NO2-B43F	9-month	RF<br>LR<br>ANN	1h	Random Forest<br>R_2: 0.89<br>RMSE: 54 ppb
Papaconstantinou et al. [7]	NO2-B43F	12-month	MEq	1h	ME: 11 ppb<br>R_2: 0.54
Bigi et al. [8]	NO2-B43F	4-month	LR<br>SVR<br>RF	1h	Zurich<br>ME: 3.2 ppb<br>R^2: 0.79<br>RMSE: 4.1 ppb<br><br>Lausanne<br>ME: 3.4 ppb<br>R^2: 0.85<br>RMSE: 4.4 ppb
Apostolopoulos et al. [9]	NO2-B43F	22-month	KNN<br>RF<br>ANN<br>LSTM<br>CNN	1h	ME: 3 ppb<br>R_2: 0.86
This study (Athens)	NO2-B43F	9-month	RF	1h	ME: 2 ppb<br>R^2: 0.7<br>nME: 0.37
This study (Patras)	NO2-B43F	9-month	RF	1h	ME: 6 ppb<br>R^2: 0.31<br>nME: 0.42
**O_3**					
Cross et al. [3]	OX-B431	4-month	HDMR	5min	ME: 7.34 ppb<br>R^2: 0.39

Zimmerman et al. [4]	OX-B431	7-month	RF	15min	R: > 0.8<br>ME: 3.4 ppb<br>nME: 0.14
Han et al. [5]	OX-B431	12-month	LSTM<br>RF	1h	Random Forest<br>R^2: 0.64<br>RMSE: 32 ppb
Papaconstantinou et al. [7]	OX-B431	12-month	MEq	1h	ME: 48 ppb<br>R_2: 0.05
Zuidema et al. [2]	OX-B431	Two-year	LR	1h	R^2: 0.81<br>RMSE: 4 ppb
Apostolopoulos et al. [9]	OX-B431	22-month	KNN<br>RF<br>ANN<br>LSTM<br>CNN	1h	ME: 4.3 ppb<br>R_2: 0.69
This study (Athens)	OX-B431	9-month	RF	1h	ME: 5.5<br>R^2: 0.78<br>nME: 0.25
This study (Patras)	OX-B431	9-month	RF	1h	ME: 10 ppb<br>R^2: 0.58<br>nME: 0.33

References

1. Ariyaratne, R.; Elangasinghe, M.A.; Zamora, M.L.; Karunaratne, D.G.G.P.; Manipura, A.; Jinadasa, K.B.S.N.; Abayalath, K.H.N. Understanding the Effect of Temperature and Relative Humidity on Sensor Sensitivities in Field Environments and Improving the Calibration Models of Multiple Electrochemical Carbon Monoxide (CO) Sensors in a Tropical Environment. *Sens. Actuators B Chem.* **2023**, *390*, 133935, doi:10.1016/j.snb.2023.133935.
2. Zuidema, C.; Afshar-Mohajer, N.; Tatum, M.; Thomas, G.; Peters, T.; Koehler, K. Efficacy of paired electrochemical sensors for measuring ozone concentrations. *J. Occup. Environ. Hyg.* **2019**, *16*, 179–190.
3. Cross, E.S.; Williams, L.R.; Lewis, D.K.; Magoon, G.R.; Onasch, T.B.; Kaminsky, M.L.; Worsnop, D.R.; Jayne, J.T. Use of Electrochemical Sensors for Measurement of Air Pollution: Correcting Interference Response and Validating Measurements. *Atmospheric Meas. Tech.* **2017**, *10*, 3575–3588, doi:10.5194/amt-10-3575-2017.
4. Zimmerman, N.; Presto, A.A.; Kumar, S.P.N.; Gu, J.; Hauryliuk, A.; Robinson, E.S.; Robinson, A.L.; R. Subramanian A Machine Learning Calibration Model Using Random Forests to Improve Sensor Performance for Lower-Cost Air Quality Monitoring. *Atmospheric Meas. Tech.* **2018**, *11*, 291–313, doi:10.5194/amt-11-291-2018.
5. Han, P.; Mei, H.; Liu, D.; Zeng, N.; Tang, X.; Wang, Y.; Pan, Y. Calibrations of Low-Cost Air Pollution Monitoring Sensors for CO, NO2, O3, and SO2. *Sensors* **2021**, *21*, 256, doi:10.3390/s21010256.
6. Vajs, I.; Drajic, D.; Gligoric, N.; Radovanovic, I.; Popovic, I. Developing Relative Humidity and Temperature Corrections for Low-Cost Sensors Using Machine Learning. *Sensors* **2021**, *21*, 3338, doi:10.3390/s21103338.
7. Papaconstantinou, R.; Demosthenous, M.; Bezantakos, S.; Hadjigeorgiou, N.; Costi, M.; Stylianou, M.; Symeou, E.; Savvides, C.; Biskos, G. Field Evaluation of Low-Cost Electrochemical Air Quality Gas Sensors under Extreme Temperature and Relative Humidity Conditions. *Atmospheric Meas. Tech.* **2023**, *16*, 3313–3329, doi:10.5194/amt-16-3313-2023.
8. Bigi, A.; Mueller, M.; Grange, S.K.; Ghermandi, G.; Hueglin, C. Performance of NO, NO₂ Low Cost Sensors and Three Calibration Approaches within a Real World Application. *Atmospheric Meas. Tech.* **2018**, *11*, 3717–3735, doi:10.5194/amt-11-3717-2018.
9. Apostolopoulos, I.D.; Fouskas, G.; Pandis, S.N. Field Calibration of a Low-Cost Air Quality Monitoring Device in an Urban Background Site Using Machine Learning Models. *Atmosphere* **2023**, *14*, 368, doi:10.3390/atmos14020368.
